# Phase-separation multiphase flow: preliminary application to analytical chemistry

**DOI:** 10.1007/s44211-023-00442-1

**Published:** 2023-10-14

**Authors:** Kazuhiko Tsukagoshi

**Affiliations:** Department of Chemical Engineering and Materials Science, Doshisha University, Kyotanabe, Kyoto 610-0321 Japan

**Keywords:** Phase-separation multiphase flow, Tube radial distribution flow (TRDF), Tube radial distribution chromatography (TRDC), Tube radial distribution extraction (TRDE), Tube radial distribution reaction (TRDR), Tube radial distribution mixing (TRDM), Phase-separation mode, High-performance liquid chromatography (HPLC)

## Abstract

A two-phase separation mixed solution can undergo phase separation from one phase to two phases (i.e., upper and lower phases) in a batch vessel in response to changes in temperature and/or pressure. This phase separation is reversible. When the mixed solution undergoes a phase change while being fed into a microspace region, a dynamic liquid–liquid interface is formed, leading to a multiphase structure. This flow is called a phase-separation multiphase flow. Annular flow in a microspace, which is one such phase-separation multiphase flow, is interesting and has been applied to chromatography, extraction, reaction fields, and mixing. Here, research papers related to phase-separation multiphase flows—ranging from the discovery of the phenomenon to basic and technical research from the viewpoint of analytical science—are reviewed. In addition, the development of a new separation mode in a high-performance liquid chromatography system based on phase-separation multiphase flow is introduced.

**Graphical abstract:**

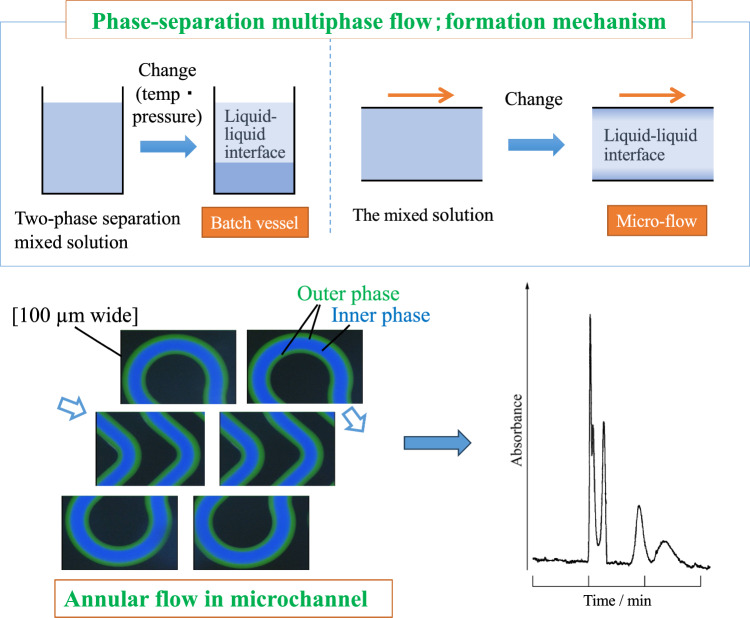

**Supplementary Information:**

The online version contains supplementary material available at 10.1007/s44211-023-00442-1.

## Introduction

### Micro-space flow in nature

Flows in channels with inner diameters of several tens to hundreds of micrometers, including channels with diameters similar to the thickness of a human hair (approximately 80 μm), are generally known as microspace flows. If we consider the flow in a microspace from a familiar, historical, and global scale, it is connected to the flow of blood in animals and the flow of water and nutrients in plants [1, 2]. Such flow in animals and plants is rarely conscious in daily life, but it plays important roles in transport and circulation functions that sustain life. Mammals and plants appeared hundreds of millions of years ago. Amazingly, living organisms have long controlled microspace flows. In addition, the improvement and development of microspace flow have accompanied the process of evolution. The flow in the bodies of animals and plants has sophisticated and advanced functions. From an engineering perspective, research on microfluidic flow is historically shallow, only being pursued since the nineteenth century. The microspace-flow-related knowledge and technology that humans can manipulate artificially are extremely limited. However, from a different viewpoint, microspace flow is an attractive area for future research.

### Electroosmotic flow, laminar flow, and separation technology

Electroosmotic flow [3, 4] and laminar flow [5] are representative flows in pipes and have been studied since the 1800s. Laminar flow features a velocity distribution different from turbulent flow [6]. In the latter half of the 1900s, a new separation technique that utilizes flow in a microspace was developed. More than 100 years passed following the discovery of microspace flow to the development of microspace-flow-related technology. For example, electroosmotic flow, along with the phenomenon of electrophoresis, gave rise to separation techniques such as capillary electrophoresis [7], micellar electrokinetic chromatography [8], and capillary electrochromatography [9]. In addition, laminar flow has led to the development of separation methods such as hydrodynamic chromatography [10], wide-bore hydrodynamic chromatography [11], and field-flow fractionation chromatography [12].

### Technological innovation and immiscible multiphase flow

As we enter the twenty-first century, along with dramatic progress in engineering, fluid control, observation or measurement, and handling technology, various microspace flows have been formed. Even in the case of laminar flow, a wide variety of methods are available to transfer liquid, including pressurization and decompression. Various ingenious approaches to inducing microspace flows have been developed, such as changing the direction of flow, mixing solutions, providing barriers within a channel, and forming segments [13–15]. Among the resultant microspace flows, immiscible multiphase flows, which are flows with dynamic liquid–liquid interfaces obtained by combining immiscible solutions such as water and oil, have been used, increasing interest in liquid–liquid interfaces. Physical phenomena and chemical reactions are involved in these trends, resulting in a wide range of research areas. However, from the viewpoint of the flow itself in the microspace, although some turbulent flow can occur, it is fundamentally a combination of electroosmotic flow, laminar flow, and immiscible multiphase flow.

### Discovery of phase-separation multiphase flow

Since 2009, our group has discovered and reported specific flows in microspaces [16–20]. Unlike immiscible multiphase flow, the flow in a microspace has a new dynamic liquid–liquid interface; research results have already been published in reviews [21–23]. In 2015, we clarified the mechanism of dynamic liquid–liquid interface formation and named it phase-separation multiphase flow [23]; we have since been conducting extensive related research [24]. Phase-separation multiphase flow is positioned as a novel flow with a dynamic liquid–liquid interface in its formation mechanism, unlike immiscible multiphase flow.

## Basic research on phase-separation multiphase flow

### Formation mechanism for phase-separation multiphase flow using two-phase separation mixed solution

**Fig. 1:**
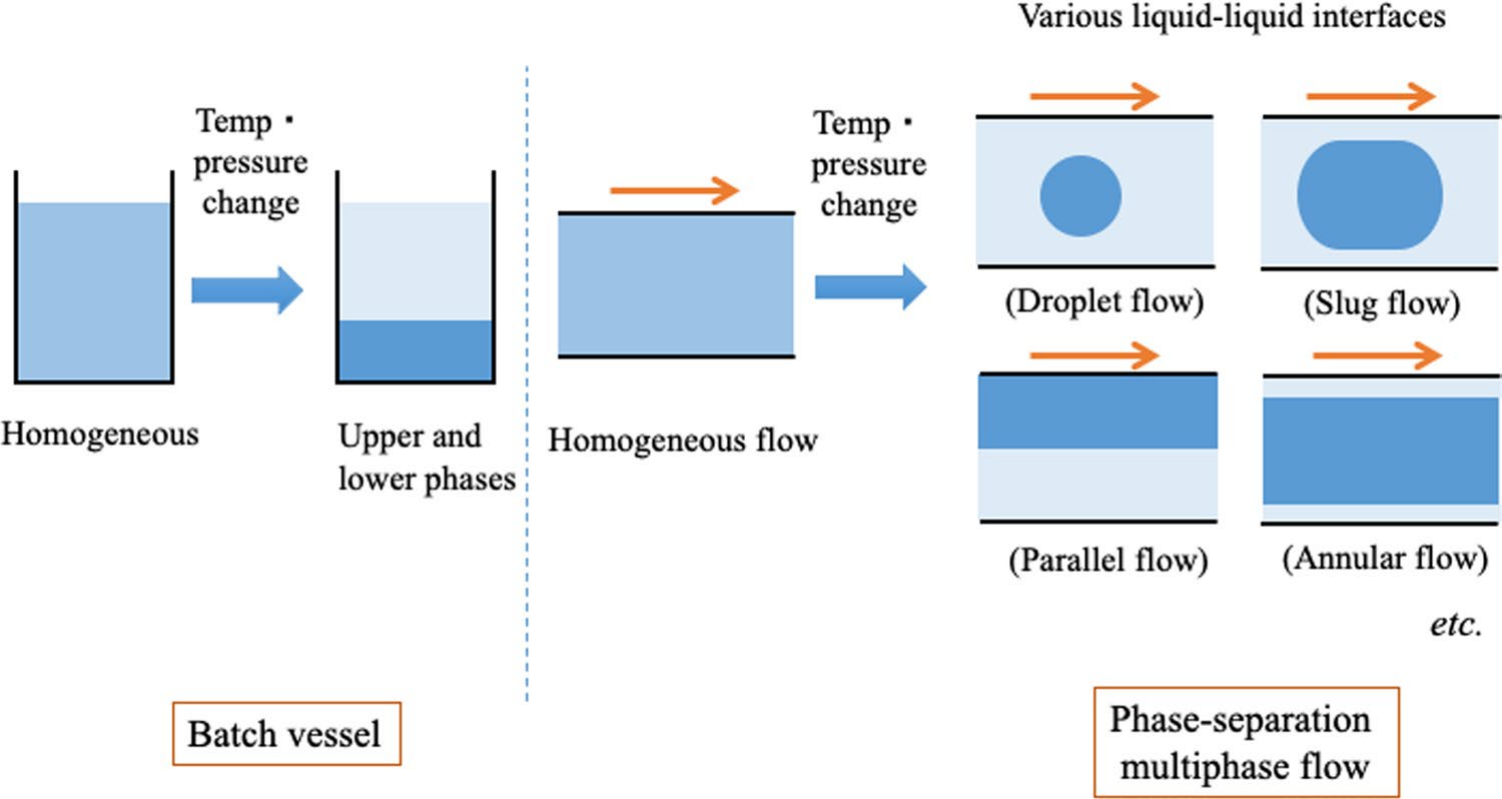
Phase-separation multiphase flow [1]

Our group has been working on basic research on phase-separation multiphase flow [24]. A two-phase-separation mixed solution changes from one phase to two phases in response to changes in pressure and/or temperature, and undergoes phase separation into upper and lower phases in a batch-type vessel [25–29]. Although the mechanism for phase separation differs for each mixed solution, factors such as intermolecular forces, hydrogen bonding, and solvation are involved, and thermochemical investigations have been conducted [25–29]. This phase change is usually reversible. However, a solution flowing through a microspace is easily temperature-controlled from the outside and is susceptible to pressure loss. Therefore, when the two-phase separation mixed solution is sent to a microspace and the phase is changed via changes in temperature or pressure, a dynamic liquid–liquid interface is formed, and a multiphase flow is obtained (Fig. 1). This flow is called a phase-separation multiphase flow. The formation mechanism for a dynamic liquid–liquid interface differs completely from that for conventional multiphase flows [30–33] (i.e., immiscible mixed-phase flows), in which two immiscible liquids join in a channel to form a liquid–liquid interface. In a phase-separation multiphase flow, a liquid–liquid interface can be formed without merging the solutions and even within a single-channel system.

As a two-phase separation mixed solution, water–hydrophilic–hydrophobic organic solvent ternary mixed solution [20], water–hydrophilic organic solvent–salt mixed solution [34], water–surfactant–salt mixed solution [35], water–ionic liquid–salt mixed solution [36], fluorocarbon–hydrocarbon organic solvent mixed solution [37], and aqueous two-phase systems (ATPSs) [38–42] such as polyethylene glycol (PEG)–dextran [43], PEG–phosphate [44], and PEG–citrate [45] have been used. The formation of a phase-separation multiphase flow has been confirmed.

### Flow visualization and annular flow

Droplet, slug, parallel, or annular flow with a dynamic liquid–liquid interface can be observed in phase-separation multiphase flow, depending on the conditions (Fig. 1). In annular flow, the inner and outer phases are formed on the basis of the distribution of solvent molecules in the radial direction of the tube. This phenomenon and flow are called the tube radial distribution phenomenon (TRDP) and the tube radial distribution flow (TRDF), respectively. Annular flow due to immiscible multiphase flow has not been reported thus far in a microspace with an inner diameter of several hundred micrometers or less [46, 47]; therefore, TRDF is interesting as a characteristic flow in a microspace.

**Fig. 2:**
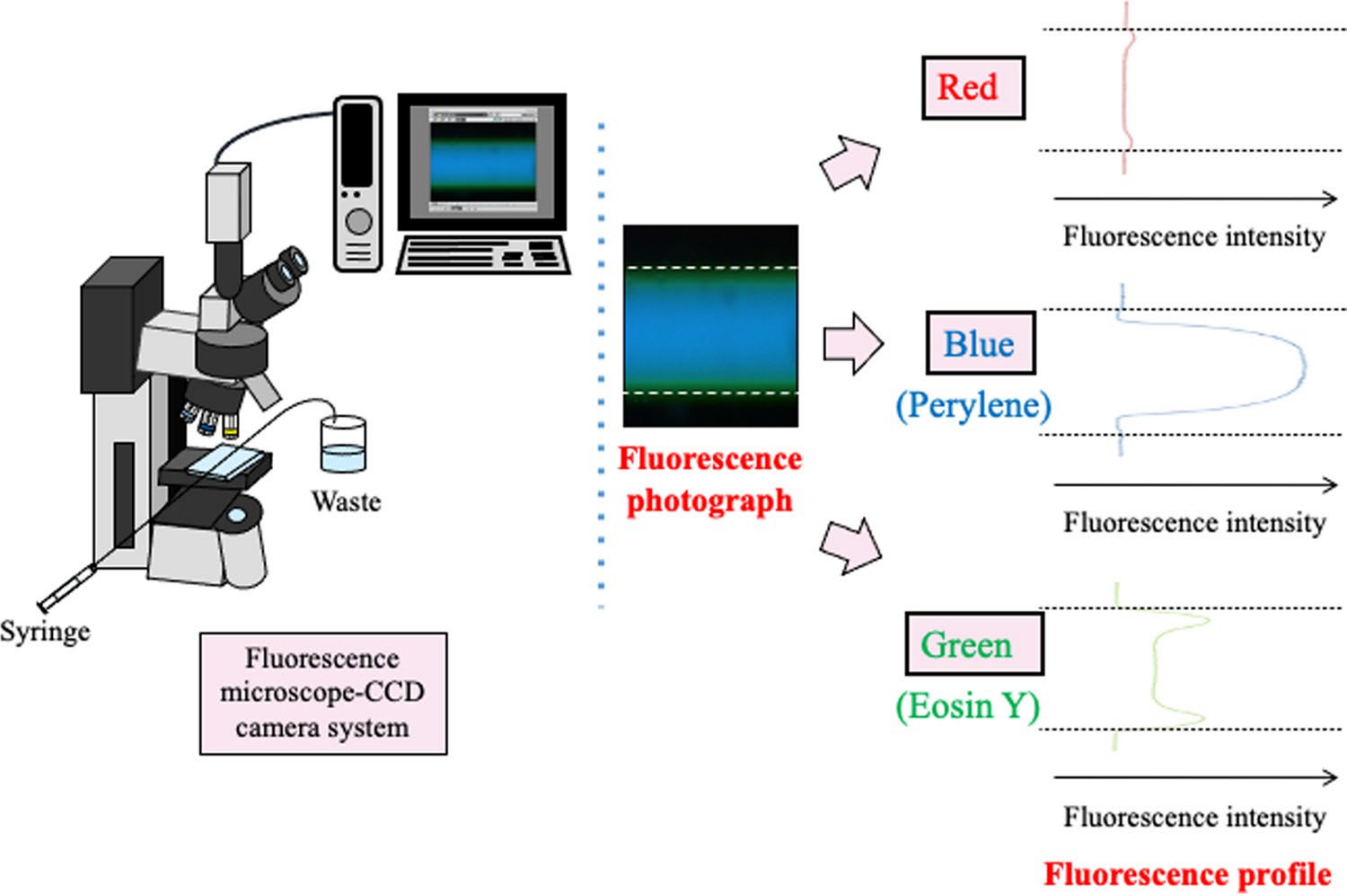
Schematic of a fluorescence microscope CCD camera and the transformation of a fluorescence photograph to a fluorescence profile through the computer. Reproduced from Ref. [21] with permission

We acquired fluorescence images of solutions flowing through microchannels or capillary tubes using a fluorescence microscope charge-coupled device (CCD) camera. We also presented the obtained fluorescence images as fluorescence profiles using image analysis software (Fig. 2). Furthermore, we observed the color of the fluorescent material in the bright field.

**Fig. 3:**
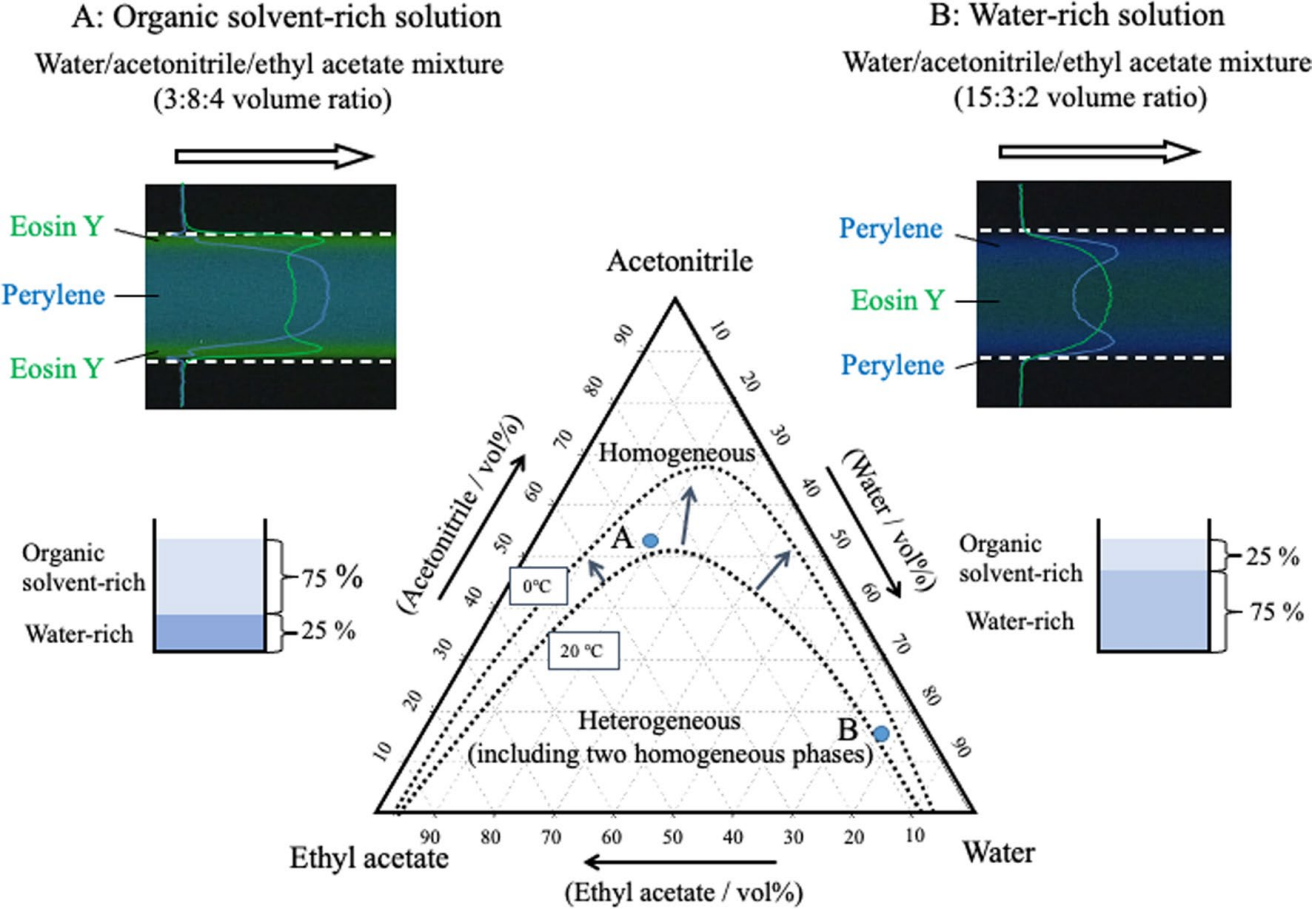
Phase diagram for water–acetonitrile–ethyl acetate mixed solution, including the solubility curves at 20 °C and 0 °C and fluorescence photographs of TRDF in the microchannel (100 µm wide). Flow rate is 1.0 μL min^−1^ [21, 24]

As an example, a TRDF fluorescence image when a water–acetonitrile–ethyl acetate ternary mixed solution containing perylene (blue) and eosin Y (green) was flowed through a microchannel is shown in Fig. 3. The composition ratio for the mixed solution was 3:8:4 for the organic solvent-rich solution and 15:3:2 for the water-rich solution. In the organic solvent-rich solution, the hydrophobic perylene was distributed in the center of the tube and the relatively hydrophilic eosin Y was distributed near the inner wall. That is, the inner phase was the organic-rich component and the outer phase was the water-rich component. However, in the water-rich solution, eosin Y was distributed in the center of the tube and perylene was distributed near the inner wall. That is, the inner phase was the water-rich component and the outer phase was the organic-rich component. TRDF could be observed irrespective of the cross-sectional shape of the microspace and the type of inner-wall material. In addition to reporting our research progress, we reported a case in which the inner-wall material affects the formation and stability of the outer phase [48]. In the formation of TRDF using a water–acetonitrile–NaCl mixed solution, the combination of an acetonitrile-rich solution and a fused silica capillary tube, as well as the combination of a water-rich solution and a polytetrafluoroethylene (PTFE) capillary tube, provided stable TRDF [34].

**Fig. 4:**
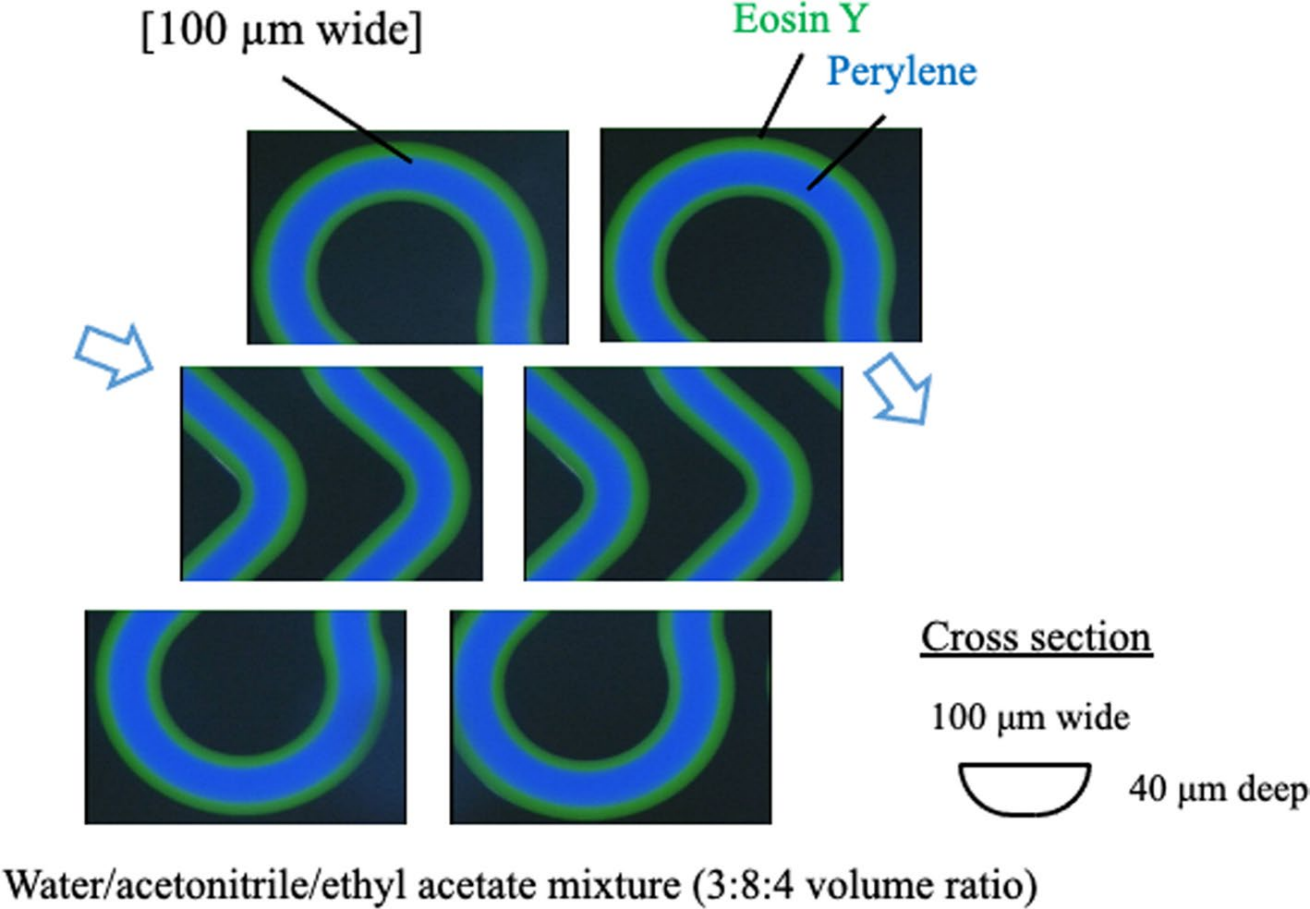
Fluorescence photographs of TRDF in the meandering microchannel. The flow rate is 2.0 μL min^−1^. Reproduced from Ref. [49] with permission

A similar experiment was performed using a three-channel convergence microchannel (Supporting Information; Fig. S1), where the organic solvent-rich solution was flowed from the three-channel side. TRDF was observed in three narrow channels. At the confluence, the outer phases coalesced to form an egg-shaped solvent puddle, which eventually broke off and diffused (Supporting Information; Fig. S2) [19]. Observation of the behavior of this fluid clearly demonstrated the formation of TRDF within the microspace. In addition, the TRDF was observed as a stable flow even in a meandering microchannel (Fig. 4) [49]. Regarding basic research on phase-separation multiphase flows, especially the formation of annular flows (TRDF), our group has carried out research focusing on the following topics.

### Construction of phase diagrams including solubility curves

A phase-separation multiphase flow is formed using a two-phase separation mixed solution. Therefore, the basic information is the phase diagram for the two-phase separation mixed solution, which includes solubility curves at different temperatures. Phase-separation multiphase flows were formed using various two-phase separation mixed solutions, and phase diagrams were constructed in all of the associated studies. The phase diagram for a water–acetonitrile–ethyl acetate ternary mixed solution is shown in Fig. 3. The solubility curves are affected by the temperature and/or pressure. The inner region of the solubility curve is the heterogeneous two-phase region, whereas the outer region is the homogeneous one-phase region. The mixed solution with composition ratios A and B is a homogeneous one-phase solution at 20 °C and a heterogeneous two-phase solution at 0 °C, respectively. In the batch-type vessel, this temperature change from 20 to 0 °C results in separation into upper and lower phases. A phase-separation multiphase flow can be attained by sending this solution into a microspace and inducing a phase change in response to changes in the temperature or pressure. Information such as the phase state, composition ratio, and temperature obtained from the phase diagram is a basic component of the study of phase-separation multiphase flow and is extremely useful. We investigated the effect of the volume ratio in greater detail by adding tie lines to the phase diagram [50].

### Considerations from viscous dissipation law and linear stability analysis

**Fig. 5:**
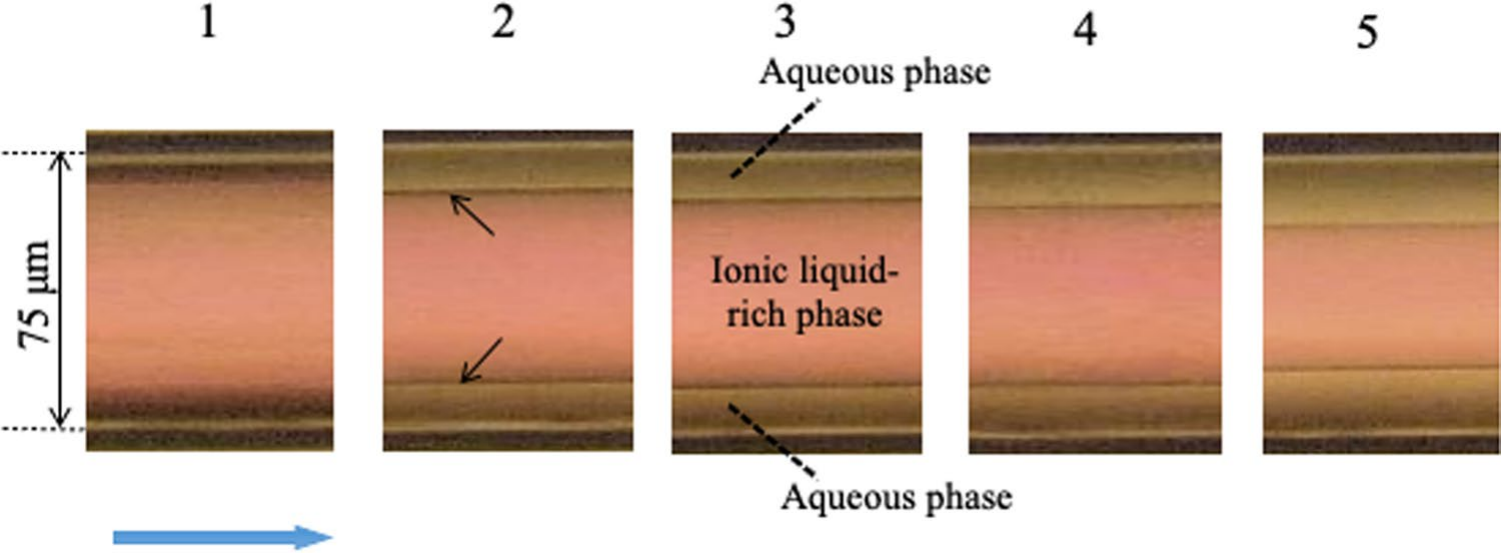
TRDF image of an ionic liquid–water mixed solution consisting of [C_4_mim]Cl and NaOH, as obtained with a bright-field microscope CCD camera system. Nos. 1–5 are as follows: (No. 1) [C_4_mim]Cl:NaOH, 48:6 w/w %; (No. 2) 32:8; (No. 3) 25:10; (No. 4) 20:13; and (No. 5) 13:20. Conditions: capillary tube, 120 cm length (effective length: 100 cm) and 75 µm i.d. fused silica; rhodamine B concentration, 5 mM; tube temperature, 15 °C; and flow rate, 1.0 µL min^−1^. Reproduced from Ref. [53] with permission

In the course of our research on TRDF, it became clear that which phase occupies the inner and outer regions is an important issue. We investigated phase diagrams for various two-phase mixed solutions, including a ternary mixed solution of water–hydrophilic–hydrophobic organic solvent [20], a water–surfactant–salt mixed solution [35], a water–ionic liquid–salt mixed solution [36], and a fluorocarbon–hydrocarbon organic solvent mixed solution [37]. Specifically, we investigated the formation of TRDF at each composition ratio, its phase configuration, and the viscosities of the inner and outer phases. When the viscosity difference between the two phases was relatively large, the phase with the higher viscosity always flowed inside without any change in phase configuration. However, when the viscosity difference was relatively small, the phase configuration depended on the volume ratio: the phase with the larger volume ratio flowed inside. In this case, the dependence of the phase configuration on the composition ratio was determined experimentally (Supporting Information; Fig. S3) [51, 52]. That is, when the composition ratio for the two-phase separation mixed solution was changed, the inner and outer phases did not change in some cases but did change in other cases. We calculated the viscous dissipation energies for five two-phase separation mixed solutions and considered the phase configuration of TRDF (Supporting Information; Eq. S1). When the viscosity difference between the two phases is large, the phase with the higher viscosity flows through the inner region according to the law of viscous dissipation [51, 52]. The formation of TRDF was investigated using 1-butyl-3-methylimidazolium chloride ([C_4_mim]Cl)/NaOH. For each composition, the volume ratio for the upper ionic liquid phase and the lower aqueous phase was varied from 9:1 to 1:9. In TRDF, the inner phase was an ionic liquid and the outer phase was a water phase. Irrespective of the volume ratio, the ionic liquid phase with high viscosity flowed inside according to the law of viscous dissipation (Fig. 5) [53].

By contrast, when the viscosity difference was small, the volume ratio became dominant and the phase with a large volume ratio flowed in the inner region. An example of a water–acetonitrile–ethyl acetate ternary system is shown in Fig. 3. This finding is consistent with the results of the linear stability analysis [19, 54]. In this case, by combining a four-way cock valve and a capillary tube, changing the composition ratio in the flow, and changing the volume ratio for both phases, we could artificially exchange the inner and outer phases [55]. However, the phase configuration in recent ATPSs such as PEG–citrate [45] and PEG–phosphate [44] cannot necessarily be explained by the viscous dissipation law and the results of linear stability analysis. Explanations of such phase configurations are left for future research.

### Consideration from the Weber number

Dimensionless numbers represented by the Reynolds number are used when discussing the flow characteristics of fluids. Using water–acetonitrile–ethyl acetate and water–acetonitrile–chloroform ternary mixed solutions, we investigated the relationship between the formation of TRDF and the Weber number [56]. The Weber number is a dimensionless number that expresses the ratio of inertial force to surface tension in fluid mechanics. The Weber numbers for the inner and outer phases were calculated for each composition ratio for the mixed solution, and the observation results of the phase-separation multiphase flow (TRDF or slug flow) were discussed together with them. When the Weber number difference between the water-rich phase and the organic solvent-rich phase is approximately equal (2 × 10^−6^ and 7 × 10^−6^ for water–acetonitrile–ethyl acetate and 4 × 10^−5^ and 5 × 10^−5^ for water–acetonitrile–chloroform), slug flow occurs. When the difference exceeds a certain value (~ 10 times difference for water–acetonitrile–ethyl acetate and ~ 3 times difference for water–acetonitrile–chloroform), TRDF is formed. On the basis of the Weber number, we inferred the possibility of TRDF formation as follows: In TRDF, the inner phase that had a larger volume ratio possessed a relatively larger Weber number than the outer phase that had a smaller volume ratio, leading to a dominant inertial force relative to the interfacial tension. Just as the Reynolds number distinguishes between the generation of laminar and turbulent flows, future investigations in various solvent systems might reveal a specific relationship between the Weber number and the generation of TRDF.

### Computer analysis

We attempted to reproduce the flow observed in TRDF by computer simulation. A two-component system of water–ethyl acetate [57] and a ternary component system of water–acetonitrile–ethyl acetate [58] were used. We here discuss the results for the ternary system. The analysis was performed using software for the volume of fluid (VOF) method and the species transport (ST) method (both Fluent programs; ANSYS, Canonsburg, USA). The VOF method is for multiphase flow analysis with a liquid–liquid interface, and the ST method is for mixed solution analysis by molecular diffusion.

**Fig. 6:**
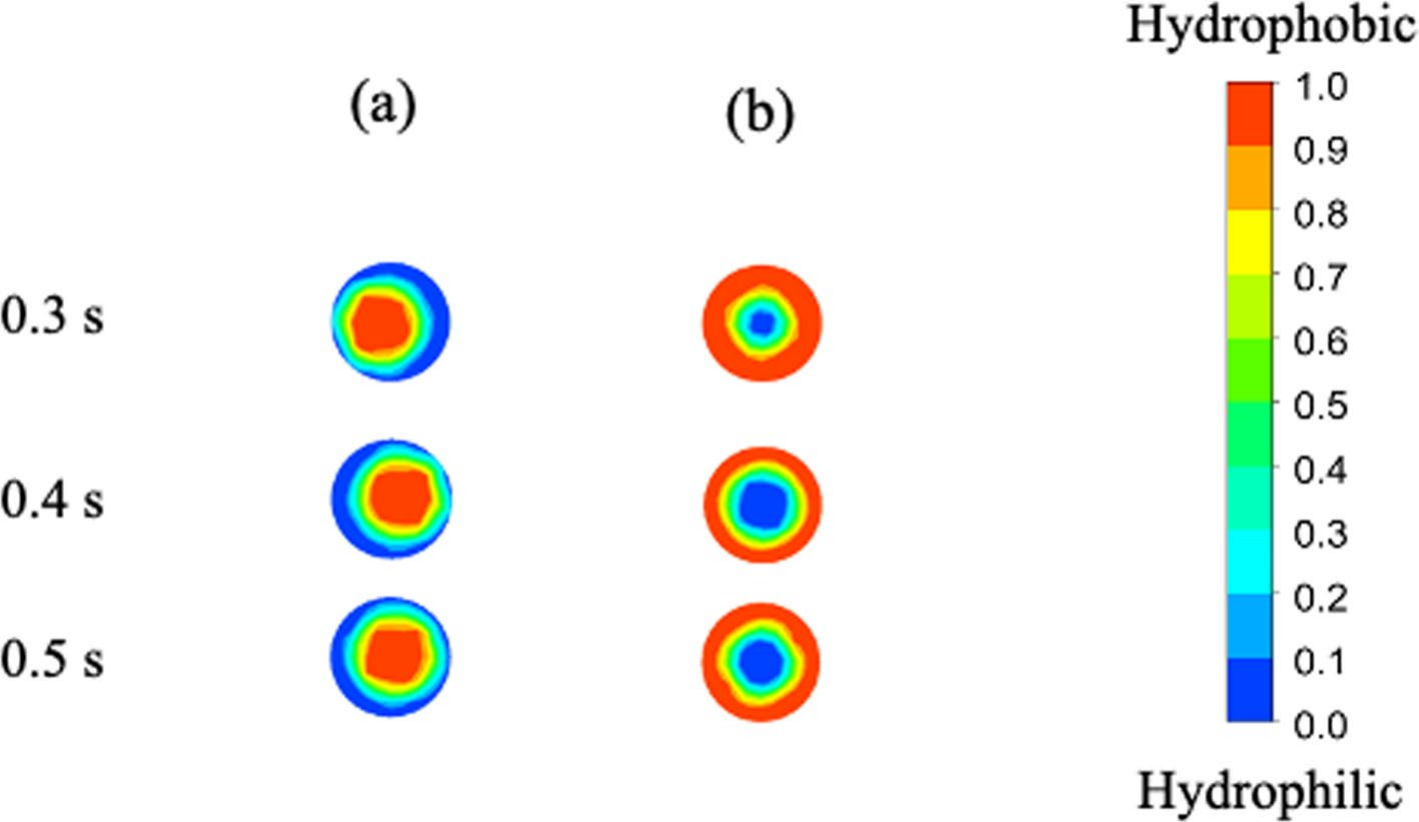
Computer simulated cross-sectional images of TRDC. **a** Water–acetonitrile–ethyl acetate mixed solution (3:8:4, volume ratio; organic solvent-rich) and **b** (4:3:1, volume ratio; water-rich). Reproduced from Ref. [58] with permission

The formation of TRDF was investigated for an organic solvent-rich composition (water–acetonitrile–ethyl acetate, 3:8:4 volume ratio) and a water-rich composition (water–acetonitrile–ethyl acetate, 4:3:1 volume ratio). Inner and outer phases could be formed at both composition ratios. Under the organic solvent-rich condition, the water-rich phase with a small volume ratio was formed as the outer phase; under the water-rich condition, the organic solvent-rich phase with a small volume ratio was formed as the outer phase (Fig. 6). In the computer simulation, both phases were exchanged depending on the solvent composition, consistent with the results of the linear stability analysis [52, 54].

## Technical research on phase-separation multiphase flow

Using TRDF, we developed technologies related to chromatography, extraction, chemical reactions, and mixing and have advanced the associated technical research [24]. These technologies are called tube radial distribution chromatography (TRDC), tube radial distribution extraction (TRDE), tube radial distribution reaction (TRDR), and tube radial distribution mixing (TRDM). For each technology, especially TRDC, we have been conducting research while being conscious of academic considerations.

### TRDC as open-tubular capillary chromatography

When both the inner and outer phases are formed by TRDF, the inner phase becomes the mobile phase, whereas the outer phase moves at a low velocity and acts as a pseudo-stationary phase to achieve chromatographic separation under laminar flow conditions [22, 23]. Academically, TRDC is classified as "open-tubular capillary chromatography." In addition to not requiring packing materials, open-tubular capillary chromatography has advantages such as a short analysis time, small solvent and sample amounts, and no need for column washing or initialization. In 2019, a critical review [59] was published. Open-tubular capillary chromatography systems are roughly divided into two types: (1) those with a tube with an inner diameter of 5 μm or less and with a sufficiently small diffusion distance of the solute and (2) those with a tube with an inner diameter of several tens of micrometers, in which case the inner wall is modified with a polymer or similar substance and a functional group that interacts with the solute is introduced (i.e., an inner-wall-modified capillary tube is used). The TRDC developed by our group does not belong to either category. In the TRDC developed by our group, a fused silica capillary tube with an inner diameter of 75–100 μm is basically used unmodified, overturning the concept of open-tubular capillary chromatography.

### TRDC system and the ternary mixed solution as an eluent

**Fig. 7:**
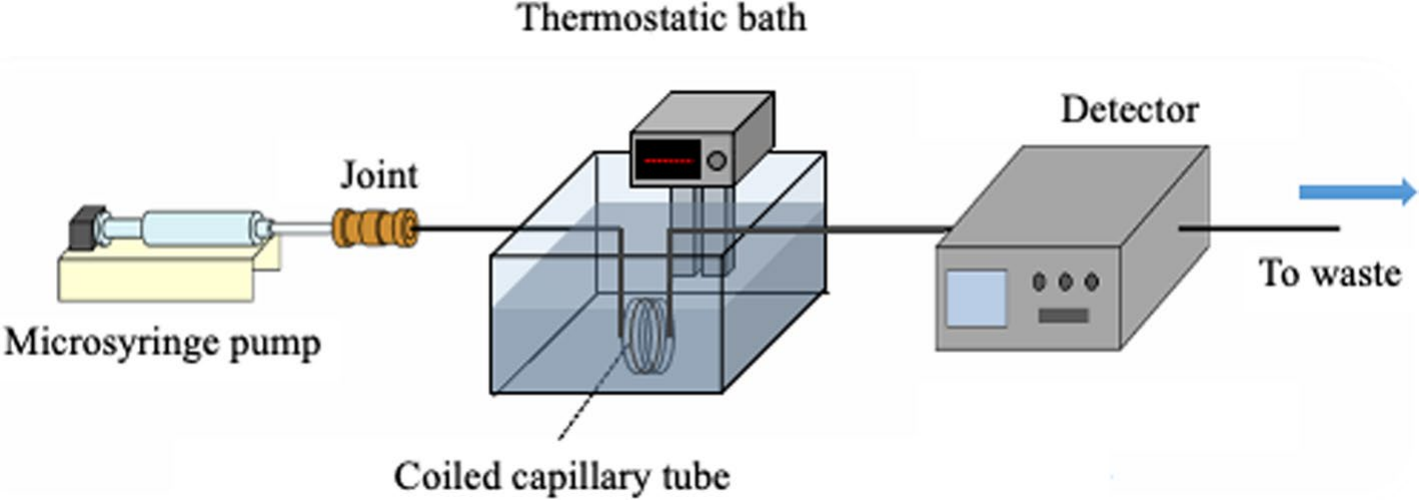
Schematic of an open-tubular capillary chromatography (TRDC system). Reproduced from Ref. [21] with permission

The homemade TRDC system mainly consists of a microsyringe pump, capillary tube, constant-temperature bath, and detector (Fig. 7) [21–23]. Fused silica, polyethylene, PTFE, and a copolymer (PTFE–perfluoroalkoxyethylene) [17, 48, 60] were used for capillary tubes. Absorption [21–23], fluorescence [61], and chemiluminescence [62, 63] were used for detection. The sample was injected from the inlet side of the capillary tube by the gravity method.

As water–hydrophilic–hydrophobic organic solvent ternary mixed solutions, water–acetonitrile–various hydrophobic organic solvents (ethyl acetate, hexane, 1-butanol, chloroform), and water–various hydrophilic organic solvents (acetonitrile, ethanol, methanol, 1-propanol, 1,4-dioxetane)–ethyl acetate mixed solutions were used to construct a ternary phase diagram; the separation of model mixed samples (1-naphthol (NA) and 2,6-naphthalenedisulfonic acid (NDS)) was subsequently investigated. Water–acetonitrile–ethyl acetate and water–acetonitrile–chloroform mixed solutions showed good separation performance [64]. Using other two-phase separation mixed solutions, such as water–hydrophilic organic compounds–salt mixed solutions [34], water–surfactant–salt mixed solutions [35], water–ionic liquid–salt mixed solutions [36], and fluorocarbon–hydrocarbon organic solvents [37], we could similarly form TRDF and develop TRDC. In the following subsections, we mainly report on TRDC using a water–acetonitrile–ethyl acetate mixed solution, for which we have abundant data.

### Normal and reversed-phase mode in TRDC and obtained chromatograms

**Fig. 8:**
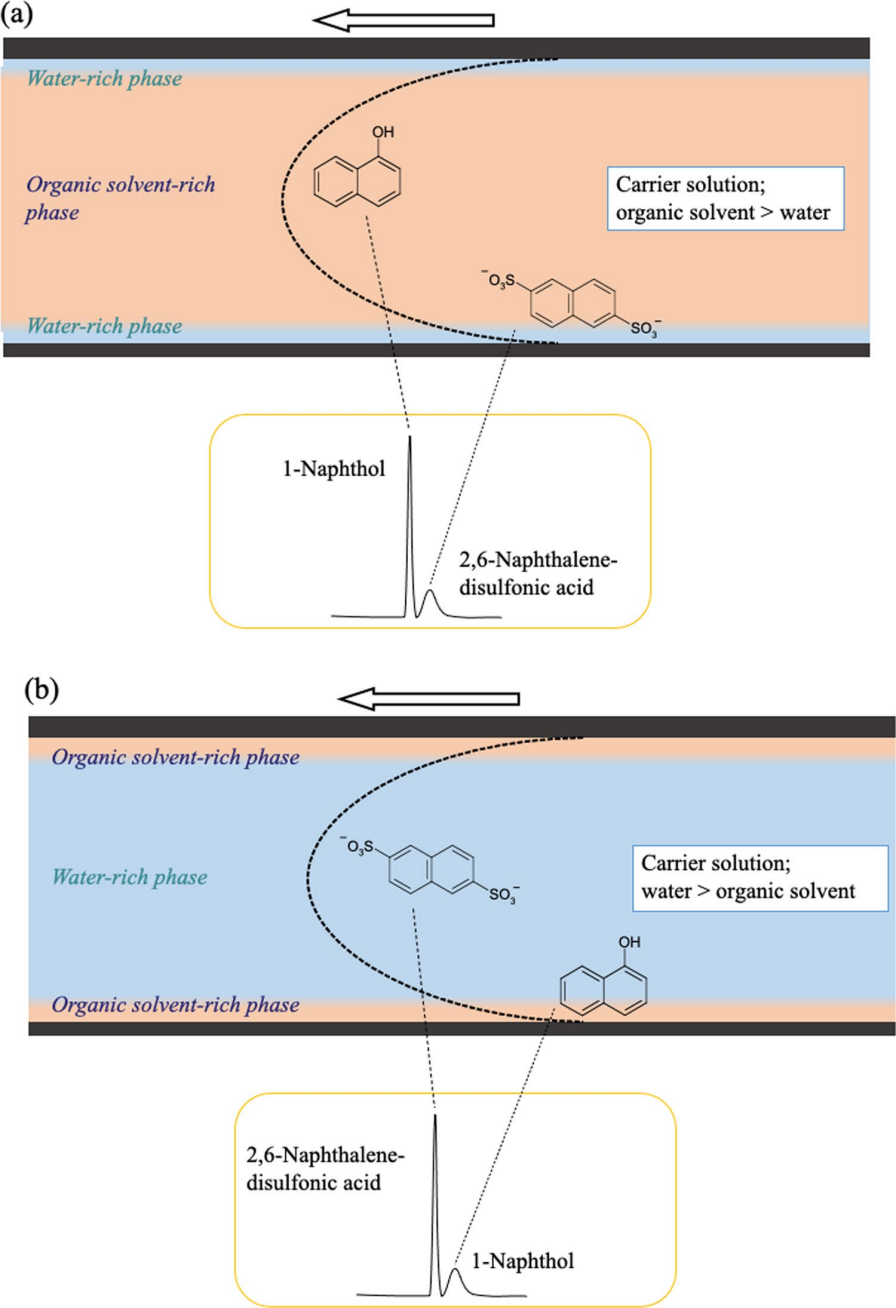
Illustration of the separation performance in a capillary tube. **a** Organic solvent-rich carrier solution and **b** water-rich carrier solution. Reproduced from Ref. [21] with permission

In a water–acetonitrile–ethyl acetate ternary mixed solution, the organic solvent-rich mixed solution (water–acetonitrile–ethyl acetate; 3:8:4 volume ratio) formed a TRDF with an organic solvent-rich inner phase and a water-rich outer phase (normal-phase mode), and the water-rich mixed solution (water–acetonitrile–ethyl acetate, 15:3:2 volume ratio) formed a TRDF with a water-rich inner phase and an organic solvent-rich outer phase (reversed-phase mode). In TRDC, the inner phase functions as the mobile phase and the outer phase functions as a pseudo-stationary phase; chromatographic separation is achieved as a result of the difference in the distribution coefficients for solutes between the two phases (Fig. 8) [21–23]. In TRDC, a normal-phase mode (Fig. 8a) and reversed-phase mode (Fig. 8b) can be formed by changing the solvent composition ratio.

In normal liquid chromatography, silica-based columns are used for normal-phase mode, and octadecyl-modified silica (ODS) columns are used for reversed-phase mode. That is, replacement of the separation column is required. In TRDC, with a change in the composition of the eluent, the inner phase (mobile phase) and outer phase (pseudo-stationary phase) can be replaced with a water-rich phase and an organic solvent-rich phase, respectively. Normal- and reversed-phase operation can be conducted properly in TRDC without exchanging the separation column (tube). This is a feature of TRDC that cannot be realized with conventional liquid chromatography.

**Fig. 9:**
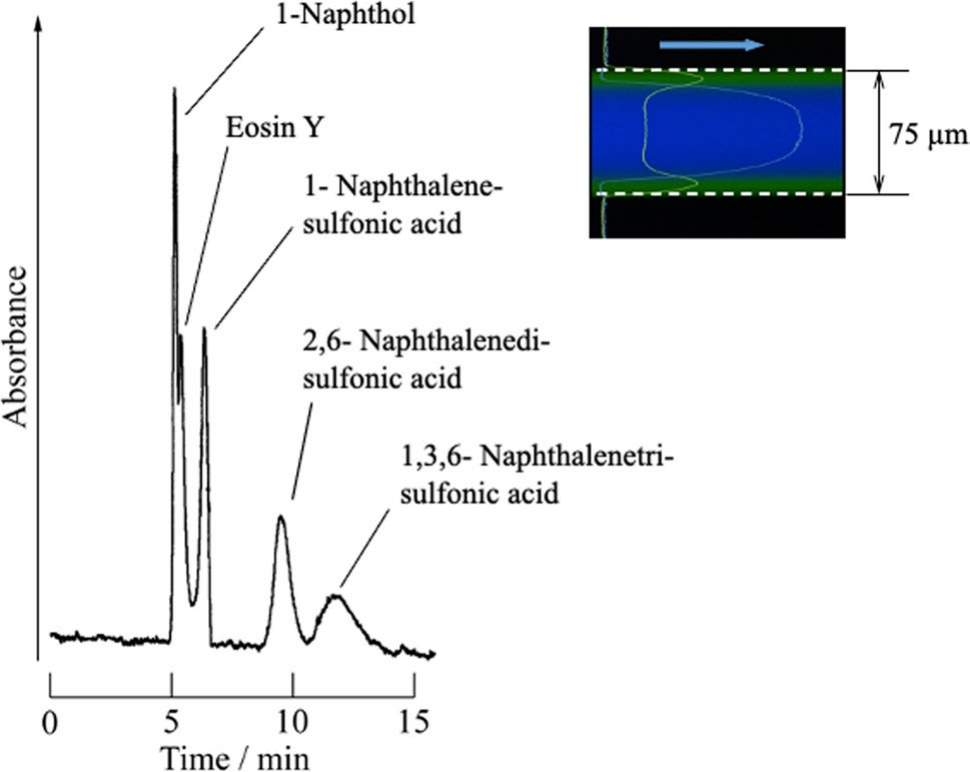
Chromatogram of a mixture analyte solution, as obtained by the TRDC system. Conditions: Capillary tube, 120 cm (effective length from the inlet: 100 cm) of 75 µm i.d. fused silica; carrier, water–acetonitrile–ethyl acetate (3:8:4 v/v/v) mixture solution; sample injection, 20 cm height (gravity) × 30 s; flow rate, 0.8 µL min^−1^; temperature, 20 °C; and 1-naphthol, 1-naphthalenesulfonic acid, and 2,6-naphthalenedisulfonic acid, 1 mM, 1,3,6-naphthalenetrisulfonic acid, 2.0 mM, and eosin Y, 0.1 mM. Reproduced from Ref. [60] with permission

The effects of the tube temperature, tube inner diameter, length, flow rate, sample volume, and solvent type on the separation performance have been investigated in detail [60, 65–69]. Because the inner diameter of the capillary tube greatly affects the back pressure, sample injection by the gravity method, and the conditions for forming TRDF, we investigated the analysis conditions using a water–acetonitrile–ethyl acetate mixed solution (organic solvent-rich solution) with an inner diameter of 75 μm [60]. 1-Naphthol, eosin Y, 1-naphthalenesulfonic acid, 2,6-naphthalenedisulfonic acid, and 1,3,6-naphthalenetrisulfonic acid were used as model mixed samples. We investigated the chromatograms of the five-component model mixture samples in normal-phase and reversed-phase operating modes [60]. In the normal-phase operation, model samples were separated and eluted in order (Fig. 9); the analytical conditions are shown in the figure caption. In reversed-phase operation, 1-naphthalenesulfonic acid, 2,6-naphthalenedisulfonic acid, and 1,3,6-naphthalenetrisulfonic acid were not separated under the experimental conditions and were eluted at almost the average linear velocity; eosin Y and 1-naphthol were thereafter detected in this order. TRDC can basically be performed using untreated open-tubular capillary tubes and mixed carrier solutions. It does not require the application of a high voltage or the use of special separation columns (i.e., packed or monolithic columns) [59].

### Application of TRDC to various samples

We have analyzed various samples using TRDC, including organic compounds, amino acids [63], peptides [63, 70], proteins [63], enzymes [63, 70], hemoproteins [71], nucleosides [72], DNA [72], metal ions [71, 73], metal complexes [71, 73], polymer microparticles [74], and optical isomers [75]. Depending on the sample, we carried out TRDC analysis by introducing a coated capillary [65, 73] or an additive [75–78]. Proteins were separated in PTFE tubes on the basis of differences in their isoelectric points using a ternary mixed solution that was water-rich [79]. Nitrate, chloride, and sulfate compounds of Ni(II), Al(III), and Fe(III) were separated by TRDC using a ternary mixed solution (water–acetonitrile–ethyl acetate) containing 8-quinolinol. The metal complexes with counter ions were successfully separated by TRDC. For example, the Ni(II) complexes eluted in the order [Ni(II)-(8-quinolinol)_3_] complex, [Ni(II)-(8-quinolinol)]-nitrate complex, [Ni(II)-(8-quinolinol)]-chloride complex, and [Ni(II)-(8-quinolinol)]-sulfate complex [80].

Isoluminol isothiocyanate (ILITC)-labeled amino acids, peptides, proteins, and enzymes were separated and detected based on their chemiluminescence [63]. Research on water–hydrophilic–hydrophobic organic solvent ternary mixed solutions and chemiluminescence revealed that the ternary mixed solution enhances the chemiluminescence of luminol (~ 6 times in intensity and ~ 8 times in lifetime enhancement compared with the case of using phosphate buffer) [81] and peroxalate esters (~ 30 times in total enhancement compared with a water–acetonitrile solution) [82].

### Considerations in TRDC

Phase diagrams, including temperature-varying solubility curves, together with tie lines drawn on phase diagrams, provided important basic information not only for the formation of phase-separation multiphase flows but also for TRDC. On the basis of this information, we investigated the conditions related to TRDC, such as the setting of the solvent composition, the phase configuration of the inner and outer phases, their flow-rate ratio, and the tendency to generate phase-separation multiphase flow [49, 83–85].

In the case of TRDC using a water–acetonitrile–ethyl acetate ternary system, we confirmed the function of the outer phase as a pseudo-stationary phase as follows. We arranged the differential equations related to the diffusion and convection of solutes in the inner and outer phases and the mass transfer at the interface (Supporting Information; Eq. S2) and derived the differential equation of the second moment [86]. We also derived a theoretical formula for the height equivalent to the theoretical plate in TRDC through the formula for dispersion (Supporting Information; Eq. S3). The theoretical value was calculated by substituting the experimental conditions and experimental data into the theoretical formula for the height equivalent to the theoretical plate. That is, the formation of inner and outer phases in TRDC and the achievement of chromatographic separation using both phases were theoretically supported [87]. In addition, we showed that the van Deemter equation holds for the relationship between the average linear velocity and the height equivalent to the theoretical plate at TRDC and considered the terms A, B, and C in the van Deemter equation at TRDC [88]. For NA, terms A and B could be ignored, whereas term C was relevant. By contrast, for NDS, term B could be ignored, whereas terms A and C were effective.

Similarly, using the water–acetonitrile–ethyl acetate ternary mixed solution, we investigated the separation behavior of the mixture of eosin Y and perylene in a capillary by fluorescence visualization. In the normal-phase mode, perylene first flowed in the center of the tube, followed by eosin Y near the inner wall (Supporting Information; Fig. S4(a)). Good agreement was attained between the elution time obtained from the fluorescence image and that obtained from the chromatogram. In addition, the average linear velocities of the inner and outer phases were calculated from the velocity distribution under laminar flow conditions, and the theoretical value of the elution time was calculated on the basis of the obtained average linear velocities. Good agreement was also observed between the theoretical and experimental elution times [89]. These findings were confirmed in the reversed-phase mode, where eosin Y flowed first in the center of the tube, followed by perylene near the inner wall (Supporting Information; Fig. S4(b)).

In addition, we compared and reported the analytical results obtained by TRDC and capillary zone electrophoresis under limited conditions [90]. In the TRDC system, the elution order of analytes could be changed by altering the component ratios of the solvents (i.e., the organic solvent-rich and the water-rich carrier solutions) within ~ 15 min. By contrast, in the CZE system, the elution order was changed by altering the electroosmotic flow direction within ~ 60 or ~ 120 min. The TRDC system could easily reverse the elution order of analytes within a short analytical time without the application of a high voltage.

We also compared TRDC and wide-bore hydrodynamic chromatography using polymer microparticles and DNA as the analysis target [91]. The elution of the microparticles resulted in a non-Gaussian peak at maximum linear velocity in wide-bore hydrodynamic chromatography but resulted in a Gaussian peak at average linear velocity in TRDC. At this time, the helix collapse of DNA was investigated for the first time using a ternary mixed solution of water–hydrophilic–hydrophobic organic solvent [92]. In addition, a slug flow that formed under a relatively wide range of conditions was found to be useful in chromatography (slug flow chromatography) because part of it functions as a pseudo-stationary phase [93].

### Microchip TRDC that matches the concept of μ-TAS

**Fig. 10:**
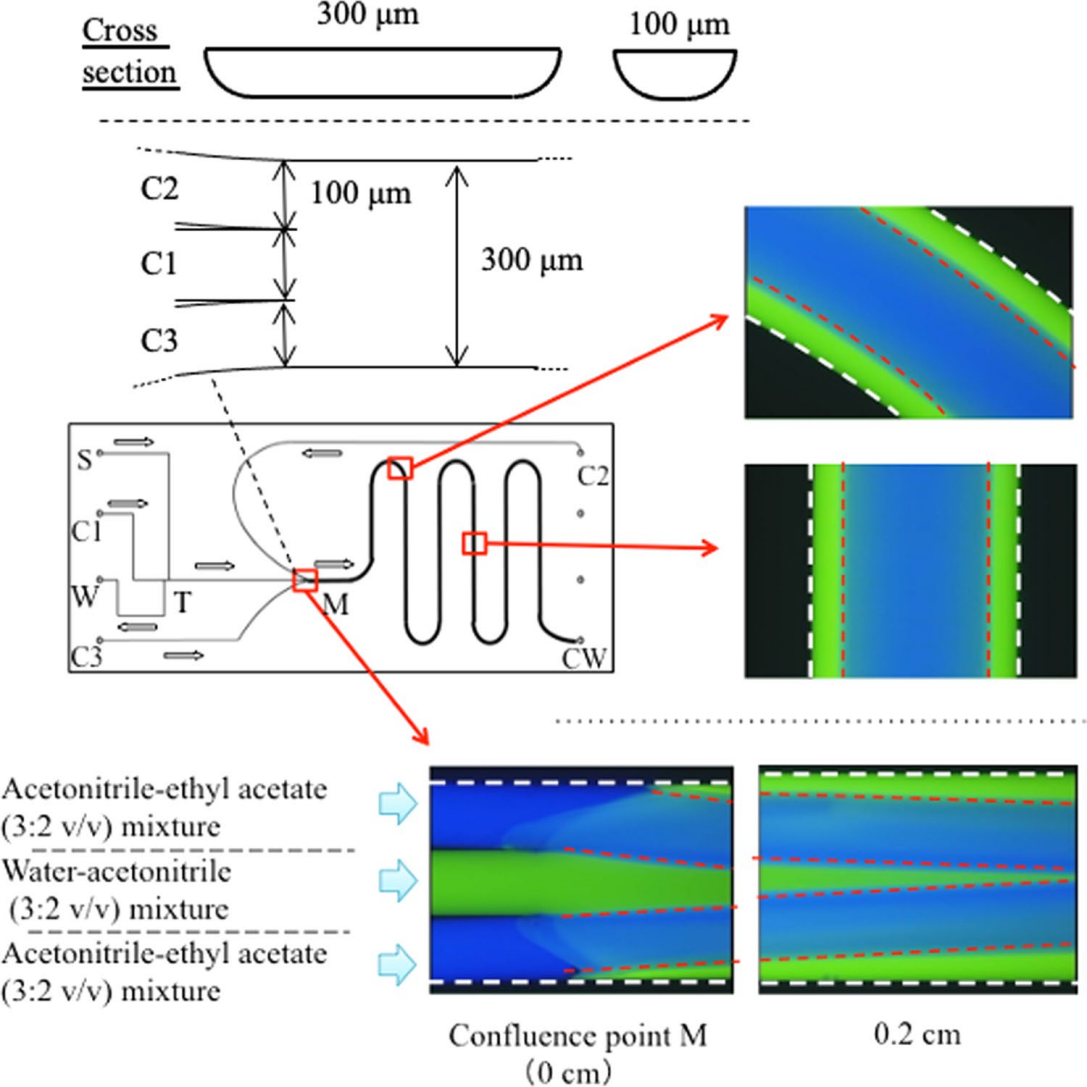
Fluorescence photographs of solvents containing dissolved fluorescence dyes at the microchannel in a microchip. Conditions: carrier for C1, water–acetonitrile (3:2 volume ratio) containing 1.0 mM eosin Y; carrier for C2 and C3, acetonitrile–ethyl acetate (3:2 volume ratio) containing 0.1 mM perylene; flow rate, 20 µL min^−1^ for C1 and 4.0 µL min^−1^ for C2 and C3; volume ratio of water–acetonitrile–ethyl acetate in the wide channel, approximately 20:53:27; temperature, 25 °C (room temperature). Reproduced from Ref. [96] with permission

We have developed a microchip-based TRDC [94, 95]. ILITC-labeled proteins were separated from free or unreacted ILITC according to the TRDC principle and detected by chemiluminescence. The microchip TRDC chemiluminescence detection system does not require a high-voltage power supply, special channel processing, light source, or spectrometer. It can be used as a separation and detection method that closely matches the concept of μ-TAS (micro-total analysis system). In addition, we have developed a microchip TRDC that forms an annular flow by TRDM instead of inducing a phase change via temperature control. The microchip and fluorescence images are shown (Fig. 10) [96].

### HP-TRDC established through application of TRDC to a commercial HPLC system

A commercial HPLC system usually consists of a plunger pump, a sample injector, a separation packed column, and an (absorption) detector. The separation packed column in a commercial HPLC system was removed, and a capillary tube was used as the separation column instead. This system is called high-performance TRDC (HP-TRDC) (Supporting Information; Fig. S5). HP-TRDC is a stepping stone for general-purpose TRDC development using a commercial HPLC system [97]. For HP-TRDC, the theoretical elution time was calculated using the average linear velocities of the inner and outer phases calculated from the velocity distribution under laminar flow conditions and the partition coefficients to both phases. Good agreement was observed between the elution time on the chromatogram and the theoretical elution time, supporting the segregation mechanism of TRDC proposed by the author [97].

In HP-TRDC, the use of a plunger pump enabled a high back pressure to be applied to the capillary tube for separation. As a result, we developed a TRDC that, unlike a device that uses a phase change induced by a temperature change, uses a phase change induced by a high back pressure without a constant temperature bath and with simplified equipment [98]. In addition, for HP-TRDC, instead of the packed column for separation, we conducted analyses using ordinary polyether ether ketone (PEEK) tubes, stainless-steel tubes [99], and capillary tubes for gas chromatography [100].

**Fig. 11:**
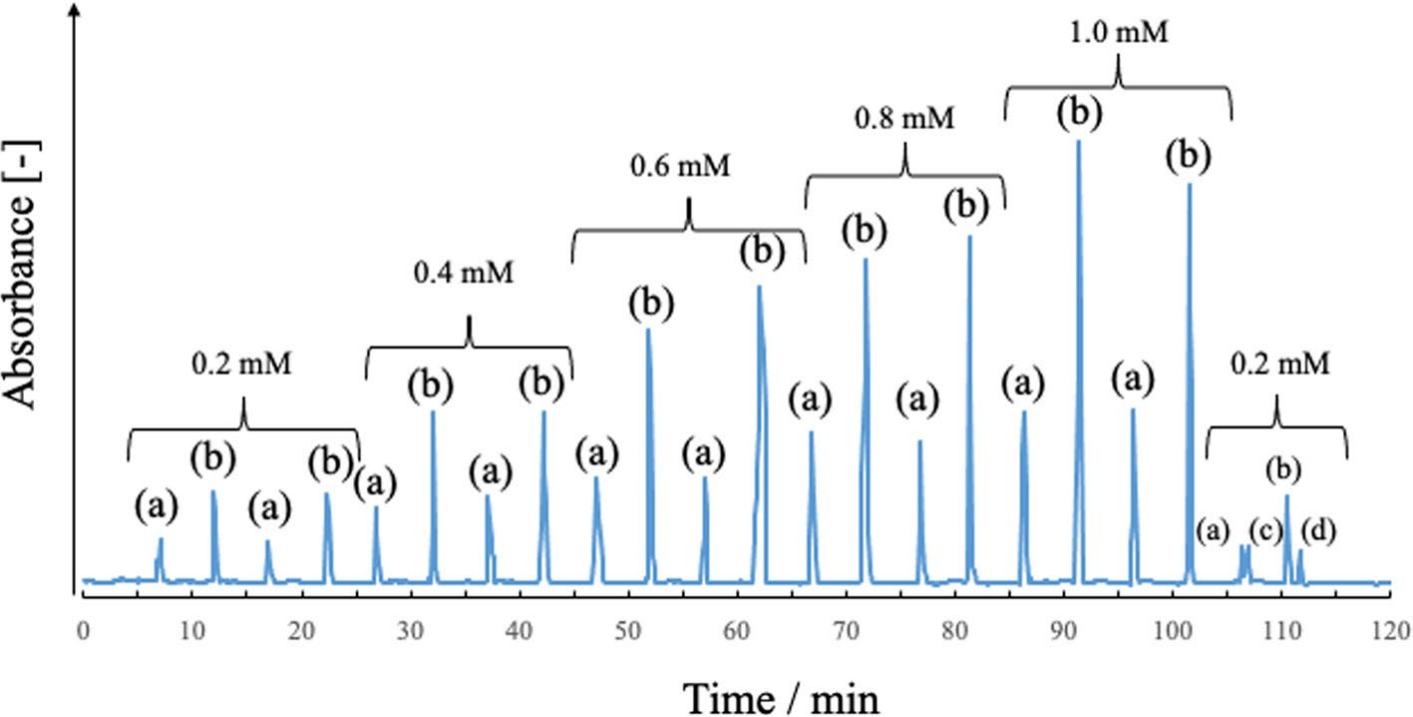
Consecutive chromatograms obtained by the TRDC system for various concentrations (two measurements) and mixed analytes (four analytes): **a** 1-naphthol, **b** 2,6-naphthalenedisulfonic acid, **c** 1-naphthalenesulfonic acid, and **d** 1,3,6-naphthalenetrisulfonic acid. Eluent, water–acetonitrile–ethyl acetate mixed solution (3:8:4, volume ratio); flow rate, 2.0 μL min^−1^; analyte injection volume, 0.2 μL; cooling temperature, 5 °C; and detection wavelength, 254 nm. Reproduced from Ref. [101] with permission

Because the separation column in TRDC is an open-tubular capillary tube and does not have a stationary phase, continuous analysis is possible without washing or initialization as a separation column. However, in the conventional homemade TRDC system, the sample solution was injected using the gravity method; continuous injection was impossible. Because HP-TRDC uses an injector to inject the sample solution, it can continuously inject the sample, unlike the gravity injection method in the homemade TRDC system. Taking advantage of these characteristics, we developed a separation and detection method for continuous sample injection using HP-TRDC (Fig. 11) [101]. We found that continuous analysis is possible with HP-TRDC and that the analysis speed was improved by approximately threefold compared with that achieved with a conventional TRDC system.

### TRDE as a microfluidic flow extraction method

Exploiting the characteristics of two-phase separation of a water–acetonitrile–ethyl acetate ternary mixed solution, we extracted metal ions in a batch process for the first time [102].

The extraction of metal ions (TRDE) was performed via TRDF formation of water–acetonitrile–ethyl acetate mixed solutions using three-way branched microchannels. TRDE is capable of continuous extraction and recovery in the stream; however, liquid transfer pumps and microfluidic devices are required. A mixed solution containing metal ions was flowed from the single-channel side, and the inner and outer phases separated into the three-channel side [103–105]. A similar extraction experiment of rhodamine B was performed using an ionic liquid instead of a ternary mixed solution [55]. Although issues such as channel design remain unsolved (microchannels are not designed for branch channel widths that match volume ratios for two-phase separations), it is interesting as an extraction method using dynamic liquid–liquid interfaces formed within microspace regions.

**Fig. 12:**
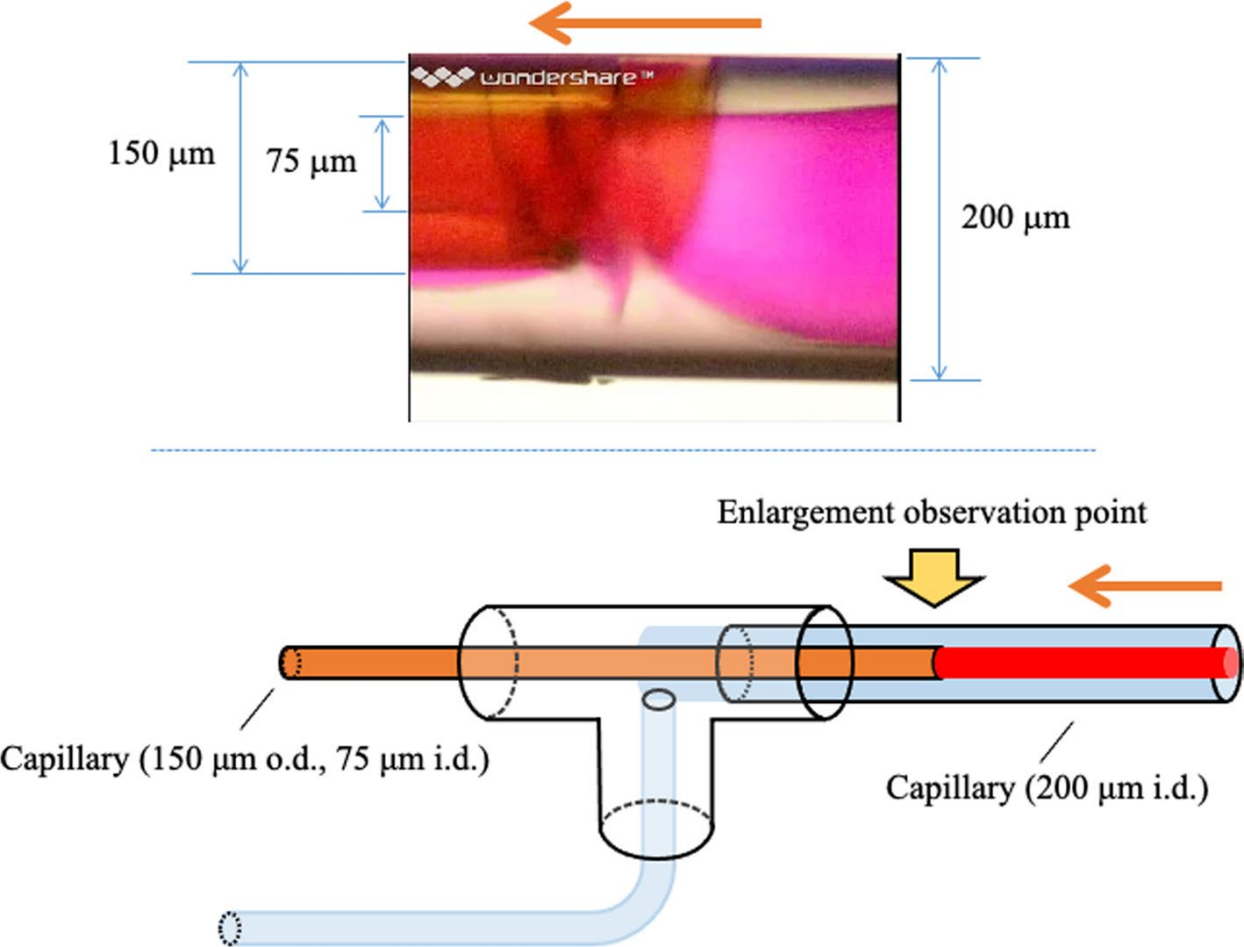
Bright-field photographs at the tip of the smaller capillary tube (75 µm i.d.) within the larger tube (200 µm i.d.) at 34 °C. The homogeneous aqueous solution containing 12 wt% Triton X-100, 2.4 M KCl, and 5 mM rhodamine B was fed at a flow rate of 20 µL min^−1^ into the larger capillary tube. Reproduced from Ref. [107] with permission

**Fig. 13:**
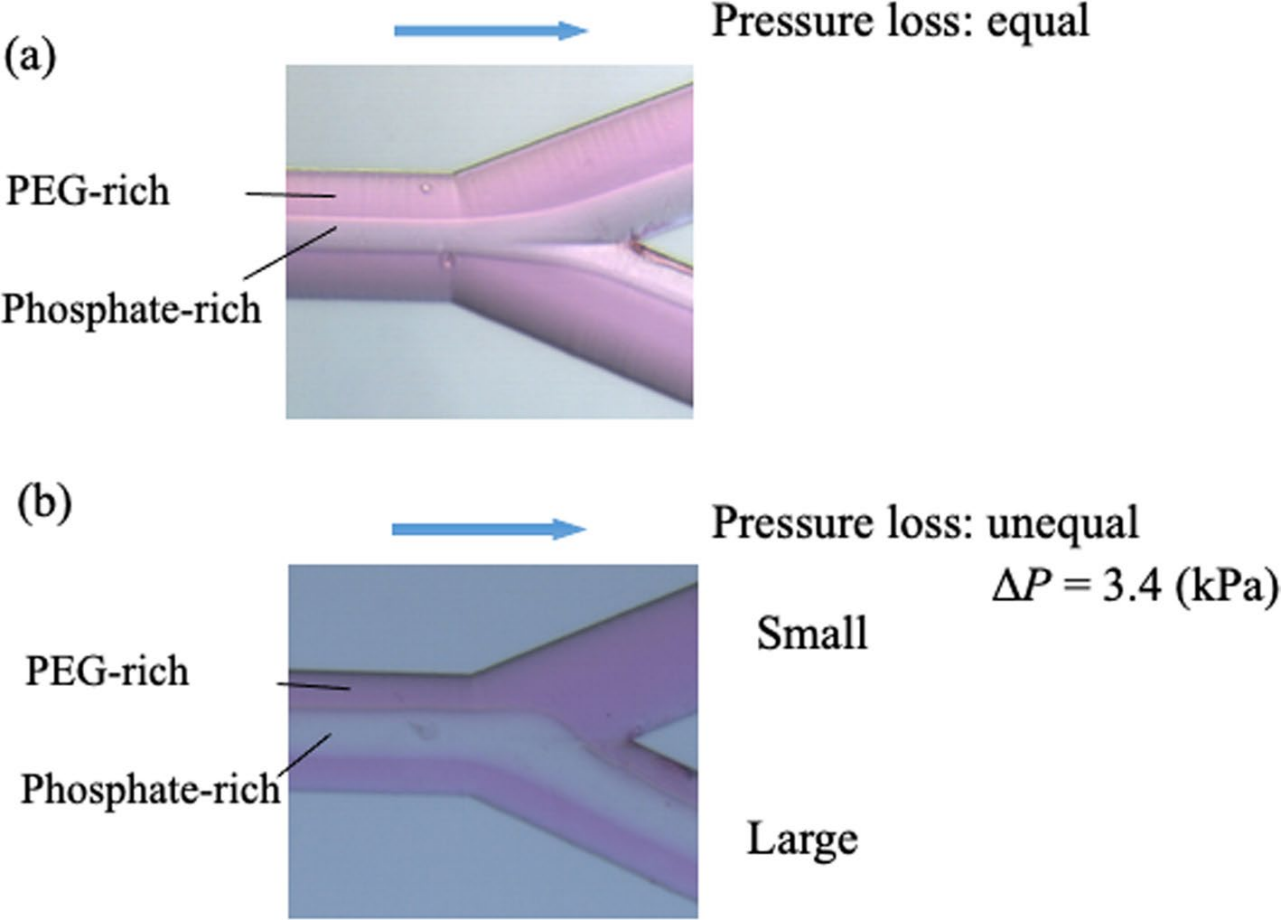
Bright-field images of microfluidic flow in a Y-type micro-channel (100 µm wide and 40 µm deep) for solution (PEG 10.0 wt% and dextran 10.0 wt%) containing 0.1 mM rhodamine B at 40 °C and at flow rate of 5.0 µL min^−1^. **a** Equal pressure loss and **b** unequal pressure loss in the separated Y-type channels. Reproduced from Ref. [45] with permission

We further assembled a double-constructed capillary tube using a T-joint [106] and performed TRDE. We used a TritonX-100–KCl mixed solution, where the inner phase was the surfactant phase and the outer phase was the water phase. When the TRDF was formed, the inner phase containing rhodamine B was collected in the capillary tube at the center of the inside (Fig. 12) [107]. In addition, protein extraction was performed using a PEG–dextran mixed solution and a double-tube capillary tube [43]. Proteins partitioned into the inner dextran phase and were collected in the inner central capillary tube. Partition equilibrium in a batch vessel required ~ 30 min; however, with TRDE occurring in a microfluidic flow, partition equilibrium was reached within a few minutes and the collection time was shortened. In addition, we proposed a new recovery method using a Y-shaped microchannel and a PEG–citrate mixed solution (Fig. 13) [45].

### TRDR that uses reaction at the kinetic liquid–liquid interface of TRDF

**Fig. 14:**
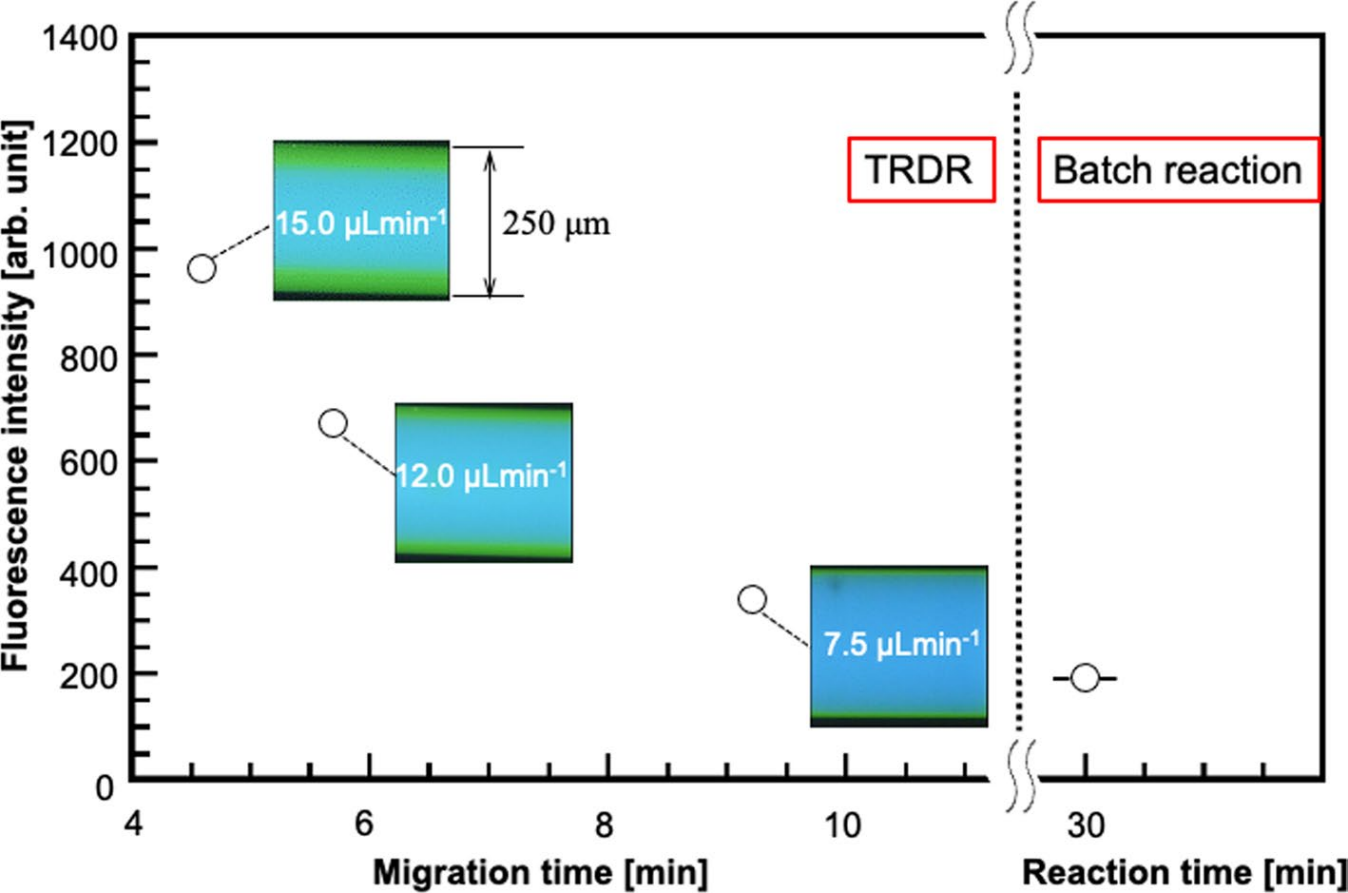
Relationship between the migration times and the fluorescence intensities in the micro-flow reaction (TRDR) system as well as between the reaction time and the fluorescence intensity in the batch reaction system [108]. The solutions included 8.3 µM BSA and 0.67 mM FR for derivatization in the systems. TRDR system (water–acetonitrile–ethyl acetate; 1:2:1 volume ratio) at 7.5, 12, and 15 µL min^−1^ and batch reaction system (water–acetonitrile; 1:2 volume ratio). Reproduced from Ref. [108] with permission

Fluorescent derivatization reactions of proteins were performed using the TRDR system with different diameter double tubes [62, 108]. The fluorescence derivatization reaction occurs at the dynamic liquid–liquid interface generated by TRDF. As a result of measuring the fluorescence of the collected reaction solution, we found that, in the TRDR system, the derivatization reaction was efficiently carried out at the liquid–liquid interface that occurred in the radial direction of the tube moving at a constant speed (Fig. 14). The specific dynamic liquid–liquid interface of TRDF served as a reaction field for TRDR and improved the reaction efficiency of fluorescence derivatization. The maximum fluorescence intensity in TRDR was ~ 5 times higher than that in a normal batch reaction.

### TRDM formed through formation of TRDF

**Fig. 15:**
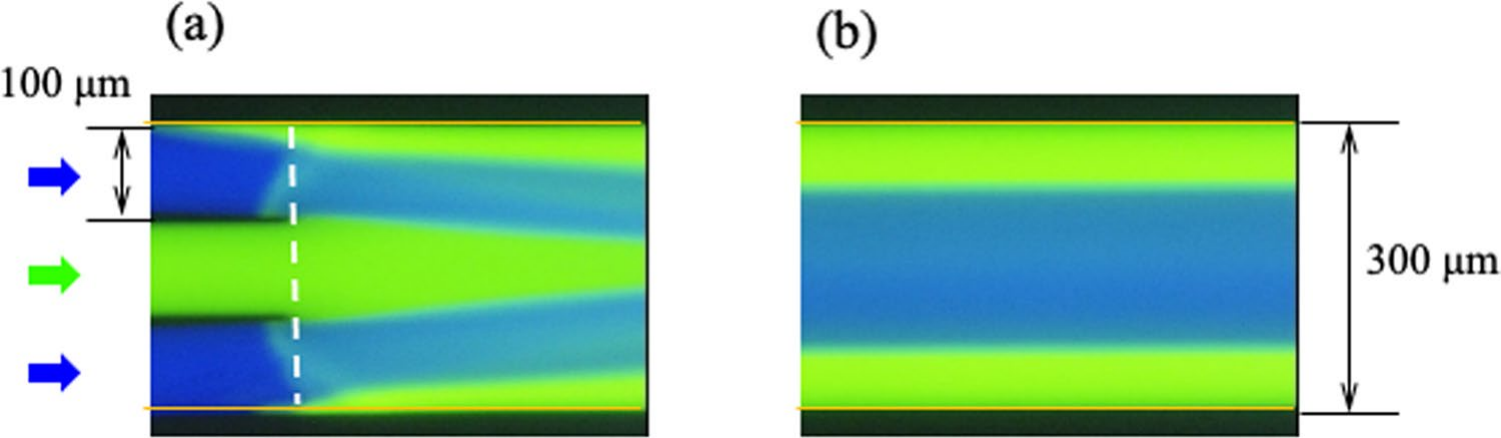
Fluorescence photographs of the solvents with the dissolved fluorescent dyes at **a** the combining point of the channels and **b** 3 cm from the combining point in a wide channel. The white dashed line indicates the combining point between the narrow channel and wide channel. Conditions: carrier, water–acetonitrile (3:2, volume ratio) containing 3.0 mM eosin Y in a center narrow channel and acetonitrile–ethyl acetate (3:2, volume ratio) containing 0.15 mM perylene in side narrow channels; flow rate, 2.0 µL min^−1^ for narrow channels. The volume ratio of water–acetonitrile–ethyl acetate in the wide channel is 3:8:4. Reproduced from Ref. [109] with permission

In TRDM, a three-way branching microchannel (Supporting Information; Fig. S1) was used from the three-way side [105]. A water–acetonitrile mixed solution containing eosin Y was passed through the center channel, and an acetonitrile–ethyl acetate mixed solution containing perylene was passed through the channels on both sides. After convergence, the flow rate was adjusted so that an organic solvent-rich solution of water–acetonitrile–ethyl acetate with a volume ratio of 3:8:4 was obtained. Fluorescence images were acquired at the confluence and downstream from the confluence. TRDF had already started to form at the confluence, and complete TRDF formation was observed 3 cm downstream (Fig. 15). During this time, the solvent underwent a special solvent mixing process [109, 110]. That is, molecular diffusion and mass transfer occurring at the dynamic liquid–liquid interface in contact with the inner wall changed the solvent composition ratio, leading to mixing [109, 110]. This mixture, TRDM, was not observed in the water–acetonitrile or water–ethyl acetate binary systems. Using a tapered microchannel [110], we were able to increase the efficiency of TRDM generation. In addition, we developed a response microfluidic analysis method using TRDM, which was combined via a four-way cock valve, to identify changes in pH and composition [110]. We also investigated TRDM using a PC simulation [110].

When a ternary mixed solution of water–hydrophilic–hydrophobic organic solvent was flowed through a Y-shaped microchannel, we found that the liquid–liquid interface was maintained but the positions of the two phases were exchanged. This flow is called microfluidic inverted flow (Supporting Information; Fig. S6) [111]. Phase control combining inverted flow and TRDF was achieved in the microchannel (Supporting Information; Fig. S7) [112].

## New adaptation of phase separation multiphase flow to a HPLC system

### A new separation mode in HPLC

The author's group used a two-phase separation mixed solution as the eluent in a HPLC system to generate a phase-separated multiphase flow within the separation column. Unlike the conventional normal-phase mode and reversed-phase mode, not only the partition of the solute between the mobile phase and the stationary phase but also the partition of the solute between the two phases of the mobile phase solution in the phase separation multiphase flow was generated. A new mode—a phase-separation mode—was proposed and implemented [113–115].

### Experimental method in phase separation mode

**Fig. 16:**
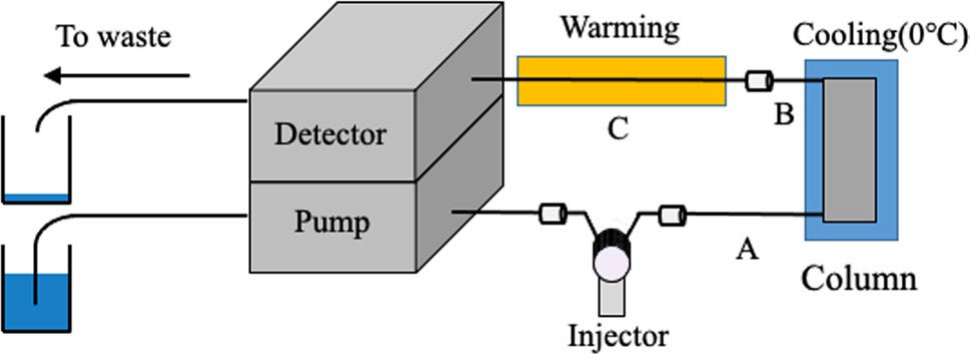
Schematic of the present HPLC system. **A** PEEK tube of 100 μm i.d. and 40 cm length; **B** PEEK tube of 130 μm i.d. and 40 cm length; and **C** fused silica capillary of 250 μm i.d. and 200 cm length. Analytical conditions for NDS and NA: eluent, water–acetonitrile–ethyl acetate mixed solution; flow rate, 100 μL min^−1^; injection volume, 20 μL; analyte concentration, 0.5 mM, and column temperature, 0 °C; and detection wavelength, 254 nm. Reproduced from Ref. [114] with permission

A commercially available HPLC system (including liquid feed pump, injector, separation column, and detector) was used (Fig. 16). As an eluent, a water–acetonitrile–ethyl acetate mixed solution, which is a two-phase separation mixed solution, was used. The column was cooled with ice water (0 °C) to induce a phase change within the column and produce a phase-separation multiphase flow. After passing through the column, the eluate was warmed and again changed in phase to form a homogeneous one-phase solution, which was sent to the detector. As separation columns, an ODS modified silica particle-packed column (ODS column) and a nonmodified silica particle-packed column (silica column) were used. ODS columns are commonly used in reversed-phase mode HPLC, and silica columns are commonly used in normal-phase mode HPLC.

### Chromatograms in reversed-phase and normal-phase modes as well as in phase-separation mode

**Fig. 17:**
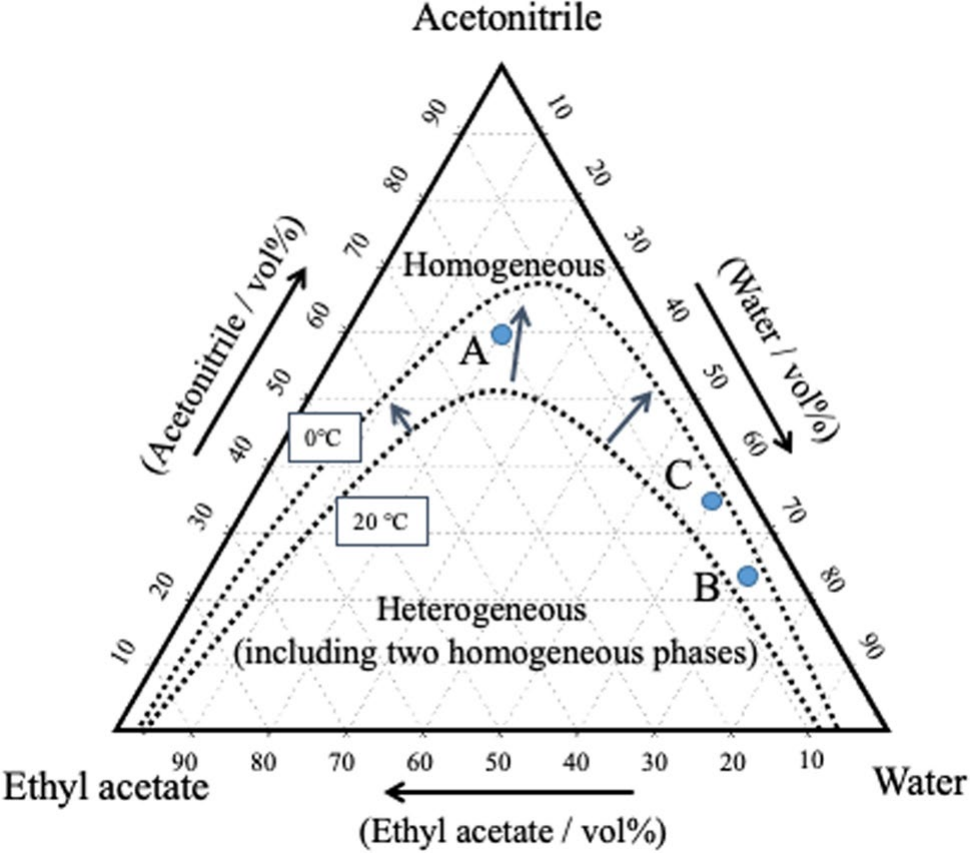
Phase diagram for water–acetonitrile–ethyl acetate mixed solution including the solubility curves at 20 °C and 0 °C. A (20:60:20, water–acetonitrile–ethyl acetate volume ratio), B (70:23:7), and C (60:35:5). Reproduced from Refs. [114, 115] with permission

**Fig. 18:**
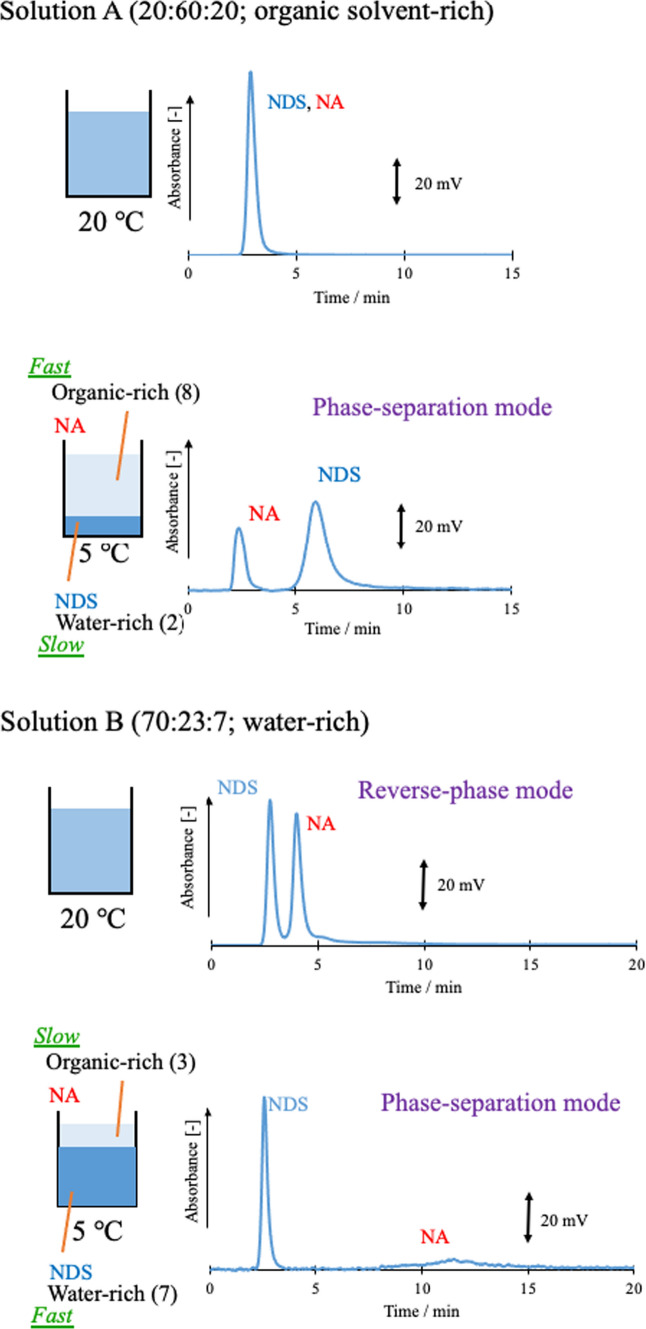
Effect of phase-separation mode on chromatograms. ODS column (Shim-pack HT-ODS; Shimadzu, Kyoto, Japan: particle type, nonporous silica base; material: octadecyl-modified silica (ODS); particle diameter, 2 µm; column size, 30 mm length × 4.6 mm inner diameter). Solution A (organic solvent-rich) and Solution B (water-rich). Reproduced from Ref. [114] with permission

First, we investigated the combination of phase-separation multiphase flow using an ODS column and a mixed solution of water–acetonitrile–ethyl acetate [113, 114]. A mixed sample of NDS and NA was used as a model sample. ODS columns are usually used in reversed-phase mode. The analytes were separated and detected in the order of NDS and NA (i.e., in reversed-phase mode). Eluent A (20:60:20) and eluent B (70:23:7) were then investigated at column temperatures of 20 °C and 0 °C. Each composition ratio is shown in the phase diagram in Fig. 17. Both eluents separated into two phases at 0 °C. The ratio of the upper phase (organic-rich phase) to the lower phase (water-rich phase) in the batch vessel was 8:2 and 3:7, respectively. At 0 °C, a phase-separated multiphase flow occurred in the column; the resultant chromatogram is shown in Fig. 18. In eluent A (20:60:20), the sample did not separate at 20 °C and eluted in the order of NA and NDS at 0 °C (i.e., in phase-separation mode). At eluent B (70:23:7) and 20 °C, the sample eluted in the order of NDS and NA (reversed-phase mode). The separation factor was greatly improved (phase-separation mode) at 0 °C.

**Fig. 19:**
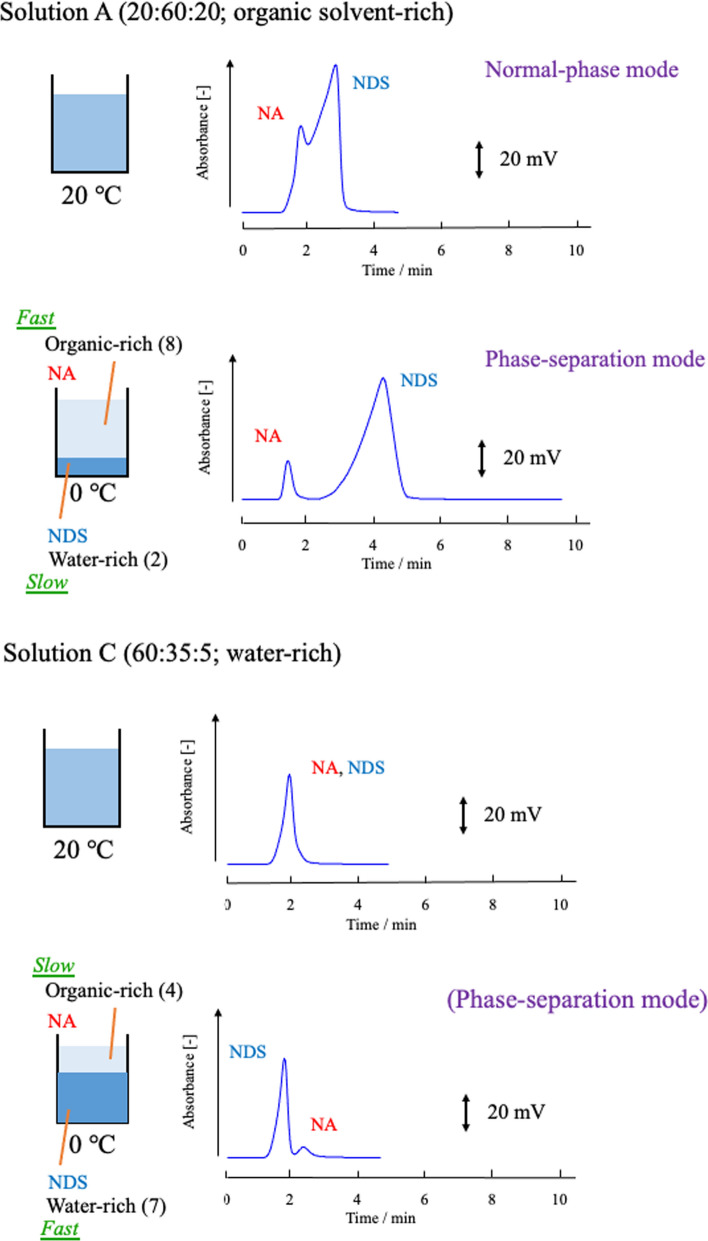
Effect of phase-separation mode on chromatograms. Silica column (Shim-pack XR-SIL; Shimadzu, Kyoto, Japan: Particle type, spherical, porous, high-purity silica particles; particle diameter, 2.2 µm; pore size, 12 nm; and column size, 50 mm length × 2.0 mm inner diameter). Solution A (organic solvent-rich) and Solution C (water-rich). Reproduced from Ref. [115] with permission

We next investigated the combination of phase-separation multiphase flow using a silica column and a mixed solution of water–acetonitrile–ethyl acetate [115]. Silica columns are typically used in normal-phase mode. For example, at room temperature (20 °C), with eluent A (20:60:20), the eluent flows through the column in a homogeneous single phase, and the sample is separated in the order of NA and NDS (i.e., in normal-phase mode). Eluent A (20:60:20) and eluent C (60:35:5) were then investigated at 20 °C and 0 °C. Each composition ratio is shown in the phase diagram (Fig. 16). Both composition ratios separate into two phases at 0 °C. The ratio of upper phase (organic-rich phase) to lower phase (water-rich phase) in the batch vessel was 8:2 and 4:7, respectively. At 0 °C, phase-separation multiphase flow occurs in the column. The resultant chromatogram is shown in Fig. 19.

In eluent A (20:60:20), at 20 °C, the sample eluted in the order of NA and NDS (normal-phase mode); at 0 °C, the elution order remained the same and the separation was greatly improved (phase-separation mode). In eluent C (60:35:5), the sample was not separated at 20 °C; however, it was eluted and separated at 0 °C in the order of NDS and NA (phase-separation mode).

### Separation mechanism for phase-separation mode

**Fig. 20:**
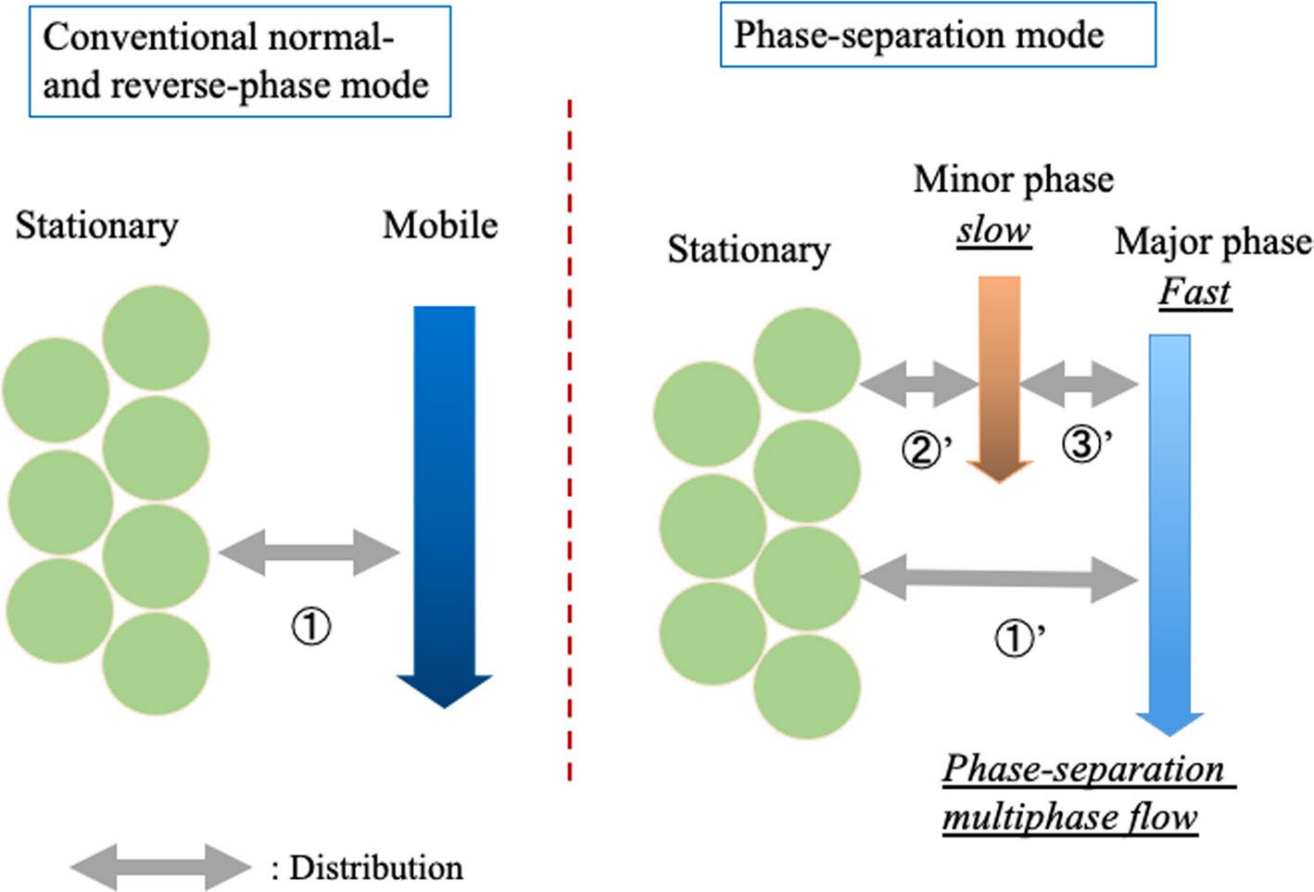
Images of the separation mechanism of the phase-separation mode and the conventional normal- and reversed-phase modes in HPLC. Reproduced from Ref. [115] with permission

Figure 20 shows the separation mechanism for phase-separation mode in HPLC and that for conventional normal- or reversed-phase modes. The conventional normal- and reversed-phase modes separate analytes on the basis of the distribution between the mobile phase and the stationary phase. On the basis of the results obtained thus far [113–115], we propose the following separation mechanism for the phase-separation mode. When the phase-separation multiphase flow is introduced as an eluent or mobile phase into the HPLC separation column, the mobile phase is separated into two phases: a major phase that moves faster and mainly far from the particle surface and a minor phase that moves slower and mainly near the surface. In this case, separation occurs (1) between the mobile phase (organic solvent-rich or water-rich phase) and the stationary phase and (2) between the two mobile phases (organic solvent-rich and water-rich phases). For example, we propose that distribution ① worked in Fig. 17B at 20 °C and Fig. 18A at 20 °C and that distributions ①', ②', and ③' worked in Fig. 17A at 0 °C, Fig. 17B at 0 °C, Fig. 18A at 0 °C and in Fig. 18C at 0 °C. No distribution worked in Fig. 17A at 20 °C or Fig. 18C at 20 °C.

We continue to research phase-separation modes in HPLC using other separation columns (e.g., a porous ODS column and core–shell silica column) and other two-phase separation mixed solutions (e.g., a water–acetonitrile–NaCl mixed solution).

## Conclusion

Flow in a microspace can occur as laminar flow and/or electroosmotic flow. An immiscible multiphase flow is a flow with a dynamic liquid–liquid interface. Thus far, microfluidic flow has been achieved through a combination of laminar, electroosmotic, and immiscible multiphase flow. Using a two-phase separation mixed solution, our group discovered a flow with a new dynamic liquid–liquid interface, which is different from the immiscible multiphase flow (i.e., a phase-separation multiphase flow). Various dynamic liquid–liquid interfaces are observed in the phase-separation multiphase flow; however, the annular flow (TRDF) is interesting because it is formed for the first time in a microspace by phase-separation multiphase flow. The formation mechanism of phase-separation multiphase flow was explored from an academic perspective. In addition, using the phase-separation multiphase flow, we developed technologies related to chromatography, extraction, chemical reactions, and mixing and systematized them. We further proposed a new separation mode for HPLC using the phase-separation multiphase flow as an eluent for a HPLC system. In the future, we expect that a wide range of research and development will be carried out from academic and technical perspectives, accompanied by the development of HPLC systems.

### Supplementary Information

Below is the link to the electronic supplementary material.

Supplementary file1 (DOCX 1018 KB)

## Data Availability

The phase-separation multiphase flow gave a useful clue to create novel analytical chemistry in a micro-space flow.
