# Supplementary material for: Phase-separation multiphase flow: preliminary application to analytical chemistry
Source: Anal Sci. 2023 Oct 14;40(1):9–28. doi: 10.1007/s44211-023-00442-1 (PMC10766728; doi:10.1007/s44211-023-00442-1)
Supplement: Supplementary file 1 — Supplementary file1 (DOCX 1018 KB) [file 44211_2023_442_MOESM1_ESM.docx]

Supporting Information


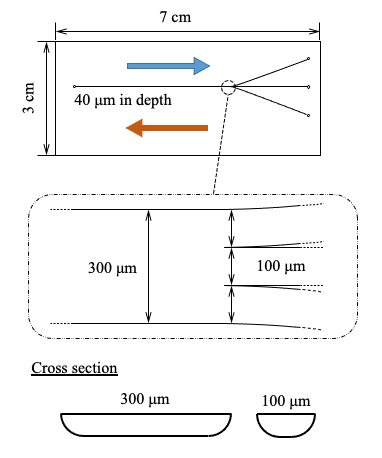


Fig. S1. Schematic of the microchip and an enlarged view of the microchip incorporating triple-branched microchannels. Reproduced from Ref. 21 with permission.


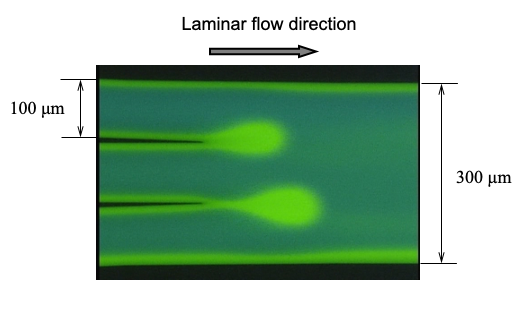


Fig. S2. Fluorescence photograph of TRDF in the microchannels (narrow channels, 100 μm wide and wide channel, 300 μm wide). Water–acetonitrile–ethyl acetate (3:8:4 volume ratio) mixture, including 0.1 mM perylene (blue) and 1 mM eosin Y (green). Flow rates are at 2.0 μL min^−1^ for the narrow channels. Reproduced from Ref. 21 with permission.


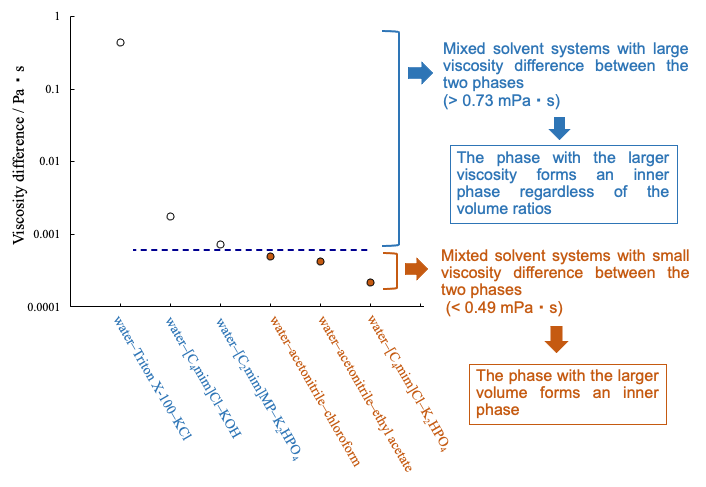


Fig. S3. Viscosity difference between the upper and lower phases for various types of two-phase separation mixed solutions from the viewpoint of inner and outer phases in TRDF [51,52]. Symbol ○: the phase with the higher viscosity formed as the inner phase irrespective of the volume ratio in TRDF. Symbol ●: the phase with the larger volume formed as the inner phase. Reproduced from Refs. 51 and 52 with permission.


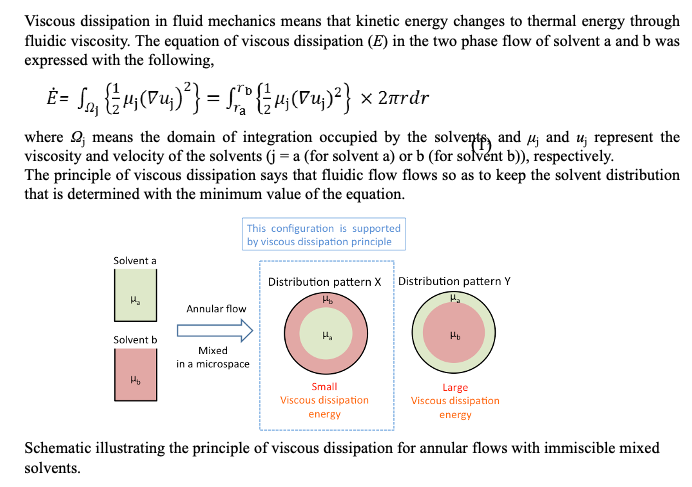


Eq. S1. The equation for viscous dissipation and the underlying concept. Reproduced from Ref. 52 with permission.


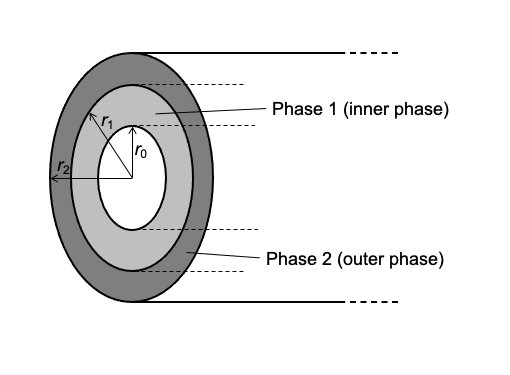


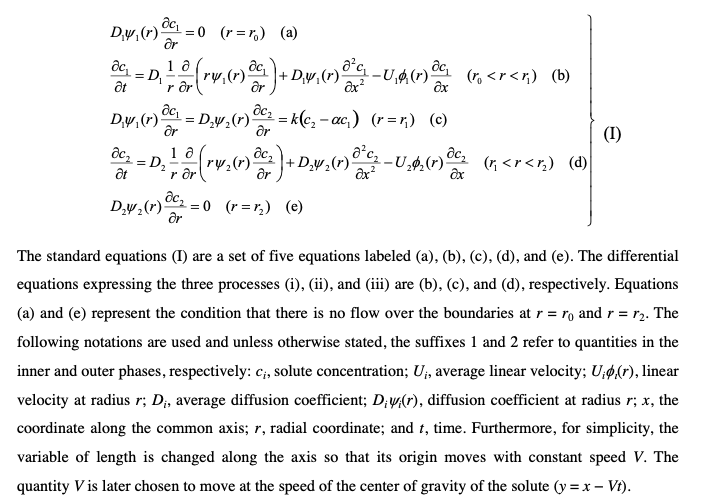


Eq. S2. The differential equations related to the diffusion and convection of solutes in the inner and outer phases and the mass transfer at the interface. Reproduced from Ref. 87 with permission.


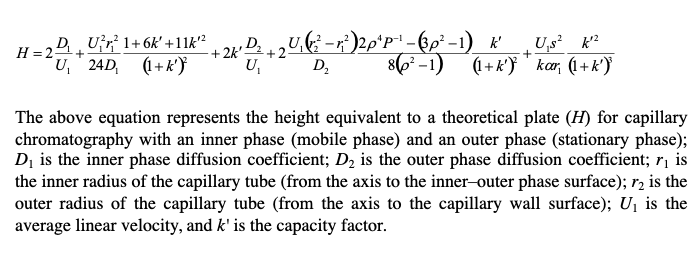


Eq. S3. Equation for the height equivalent to a theoretical plate. Reproduced from Ref. 87 with permission.


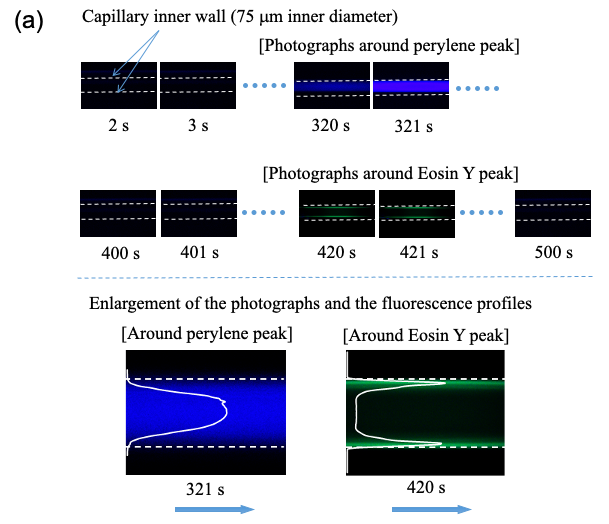


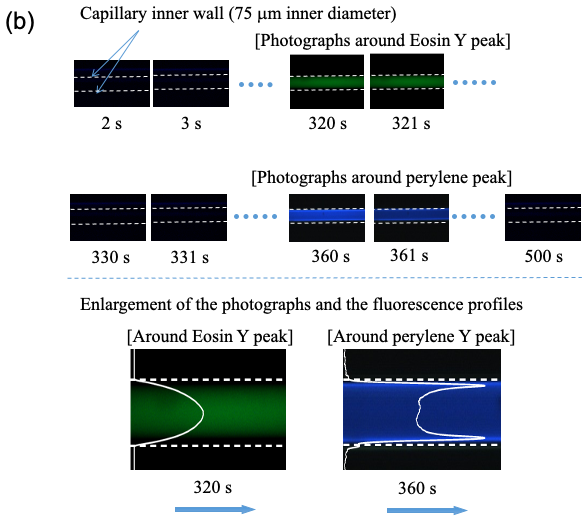


Fig. S4. Fluorescence photographs of the microfluidic behavior of the analytes, perylene (blue) and eosin Y (green), in the capillary tube with (a) the organic solvent-rich eluent solution (water–acetonitrile–ethyl acetate; 3:8:4 volume ratio) (b) the water-rich eluent solution (water–acetonitrile–ethyl acetate; 4:2:1 volume ratio). Reproduced from Ref. 89 with permission.


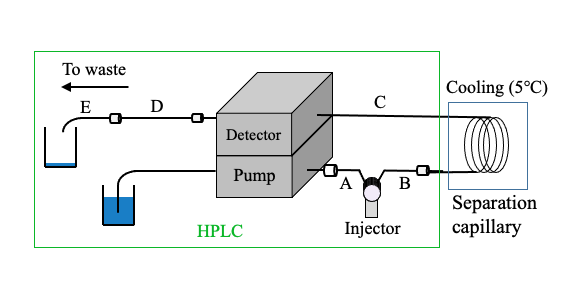


Fig. S5. Schematic of the HP TRDC system. A: PEEK tube of 130 μm i.d. and 50 cm length; B: PEEK tube of 130 μm i.d. and 10 cm length; C: (separation capillary tube), fused-silica capillary of 75 μm i.d. and 250 cm length; D: fused-silica capillary of 75 μm i.d. and 150 cm length; and E: fused-silica capillary of 50 μm i.d. and 50 cm length. Eluent, water–acetonitrile–ethyl acetate mixed solution (3:8:4, volume ratio); flow rate, 2.0 μL min^−1^; analyte injection volume, 0.2 μL; cooling temperature, 5 °C; and detection wavelength, 254 nm. Reproduced from Ref. 24 with permission.


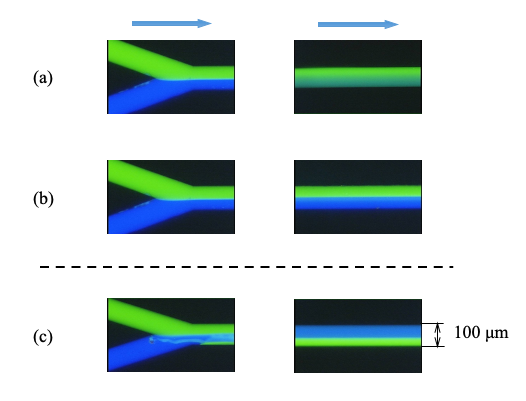


Fig. S6. Microfluidic flow in a Y-type microchannel (100 μm wide and 40 μm deep): (a) parallel-homogeneous flow, (b) parallel flow, and (c) inverted flow. Conditions: (a) water containing 1.0 mM eosin Y and acetonitrile containing 0.1 mM perylene combined at the same flow rates (total flow rate in the single channel, 10 μL min^–1^), (b) water containing 1.0 mM eosin Y and ethyl acetate containing 0.1 mM perylene combined at the same flow rates (total flow rate in the single channel, 10 μL min^–1^), and (c) water–acetonitrile (3:4.5, *v/v*) containing 1.0 mM eosin Y and acetonitrile/ethyl acetate (3.5:4, *v/v*) containing 0.1 mM perylene combined at the same flow rates (total flow rate in the single channel, 10 μL min^–1^). Fluorescence photographs were taken at the combining point and 3 cm from the combining point in the single channel. Reproduced from Ref. 111 with permission.


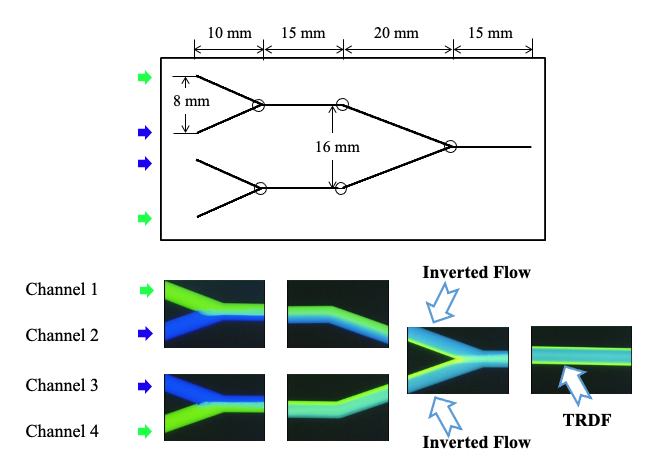


Fig. S7. Schematic of a microchip with three Y-type mixing points in the microchannel. The conditions in channels 1 and 4 are 2.0 mM eosin Y dissolved in water–acetonitrile (20:30 volume ratio); channels 2 and 3 are 0.2 mM perylene dissolved in acetonitrile–ethyl acetate (20:30 volume ratio). The flow rate is 2.0 μL min^−1^ each at 20 °C. Reproduced from Ref. 24 with permission.
